# Rapid temporal processing in the olfactory bulb underlies concentration invariant odor identification and signal decorrelation

**DOI:** 10.21203/rs.3.rs-4415331/v1

**Published:** 2025-07-04

**Authors:** Mursel Karadas, Jonathan V. Gill, Sebastian Ceballo, Shy Shoham, Dmitry Rinberg

**Affiliations:** 1 Neuroscience Institute, New York University Langone Health, New York, NY, 10016.; 2 Tech4Health Institute, New York University Langone Health, New York, NY, 10016.; 3 Department of Ophthalmology, New York University Langone Health, New York, NY, 10016.; 4 Center for Neural Science, New York University, New York, NY, 10016.

## Abstract

In a dynamic environment, sensory systems must filter out irrelevant information to construct a stable percept. Animals who rely on smell need to identify and discriminate odors despite fluctuations in concentration, yet odor receptor activation is strongly concentration-dependent. Here, we explored how odor signals are transformed within the mouse olfactory bulb (OB) by developing an all-optical approach to identify the connectivity between odor receptor channels (glomeruli) and the mitral and tufted cells (MTCs), while monitoring their odor responses. We found that the glomeruli and MTCs activated earliest in a sniff robustly represented odor identity across concentrations, while MTCs connected to later-activated glomeruli were concentration-dependent. Furthermore, probing the responsiveness of MTCs to glomerular input found a short temporal window of excitability at a sniff’s start, followed by prolonged odor-evoked inhibition. Our findings reveal a temporal filter implemented by the OB, responsible for stabilizing identity across concentrations while decorrelating responses between odors.

## Introduction

Sensation begins when stimuli from the external world interact with sensory receptors. The initial layers of neural processing transform and reformat receptor information prior to conveying it to downstream brain areas. This transformation is not arbitrary, serving specific computational functions and laying the foundation for subsequent processing. While cortical computations are thought to play key roles in object recognition and the extraction of complex features like faces^1^, early processing in areas like the retina performs crucial tasks like edge detection^2^, background movement subtraction^3^, and intensity normalization^4^. Learning the hierarchy and logic of these transformations is crucial for understanding sensory processing.

The olfactory system provides a good model for studying the principles of peripheral sensory processing due to its well-defined anatomical organization and genetic accessibility. In mouse olfaction, the panoply of chemical odorants is sensed by ~5 million olfactory sensory neurons (OSNs), whose axons are segregated into ~2,000 glomeruli based on the genetic identity of receptors in the olfactory bulb (OB). Odorants bind to unique sets of olfactory receptors and elicit specific, highly-reproducible, spatiotemporal patterns of activity across glomeruli^5–8^, which in turn activate the next layer of neurons, the Mitral and Tufted cells (MTCs)^9–12^. While the MTCs each receive their primary input from a single glomerulus, they are embedded in a vast inhibitory network of interneurons mediating lateral inhibition^13–16^ and exhibit complex temporal dynamics through which odor information is encoded on both fast^10,12^ and slow timescales^17,18^. MTCs are the only cells that send their output from the OB to downstream brain areas, thus, observing their dynamics provides important clues for understanding the pre-cortical computations of olfactory processing.

Early olfactory signal transformations (i.e., from glomeruli to the MTCs) have been proposed to underlie multiple computational roles, including lateral inhibition for contrast enhancement^19^, divisive normalization^19–22^ for concentration-invariant odor encoding^23^ and pattern decorrelation for enhancing odor discriminability^18,24,25^. However, there is little direct behavioral evidence for what role these computations play in odor identification^26^. Furthermore, these proposed computations generally require relatively long time intervals (hundreds of milliseconds to seconds), which are substantially longer than the time required for odor recognition observed in behavioral experiments (<100 ms)^8,27,28^. This leaves open the question of how the OB transforms odor activity patterns so they can be rapidly identified across a range of concentrations while keeping their identities distinct^8,26–28^.

To understand the logic and purpose of computations performed by the OB, as well as their underlying neural mechanisms, we require the ability to (i) track the temporal dynamics of a large number of glomeruli and MTCs in response to large number of odor stimuli, (ii) to establish connectivity between glomeruli and MTCs, and (iii) to perturb neural processing with high temporal resolution. In this study, we took advantage of an all-optical approach, which combines 2-photon (2P) Calcium (Ca^2+^) imaging of glomeruli and MTCs with glomerular optogenetic pattern stimulation. This approach allowed us to establish functional connectivity between glomeruli and MTCs, as well as characterize MTC responses to highly controllable synthetic and natural stimuli at timescales relevant for odor recognition.

Our results reveal a new computational role of the OB network: *temporal filtering*, by which early odor activated glomeruli can effectively drive responses in their recipient MTCs, while later activated glomeruli cannot. We demonstrate that temporal filtering has profound consequences, preventing excitatory transmission except within a short time window, effectively decoupling MTC responses from the activity of their input glomeruli. These results lead us to an integrative model connecting the odor affinity of OSNs to the lateral interactions between glomerular channels. We demonstrate that these interactions are highly temporally dependent. Finally, this model and a series of experiments help resolve previously ambiguous findings about the stereotypy and diversity of tuning across MTCs sharing the same glomerular input, and test predictions for more complex scenarios, such as predicting the non-linear responses of MTCs to mixtures of odors.

## Results

**Figure 1.**
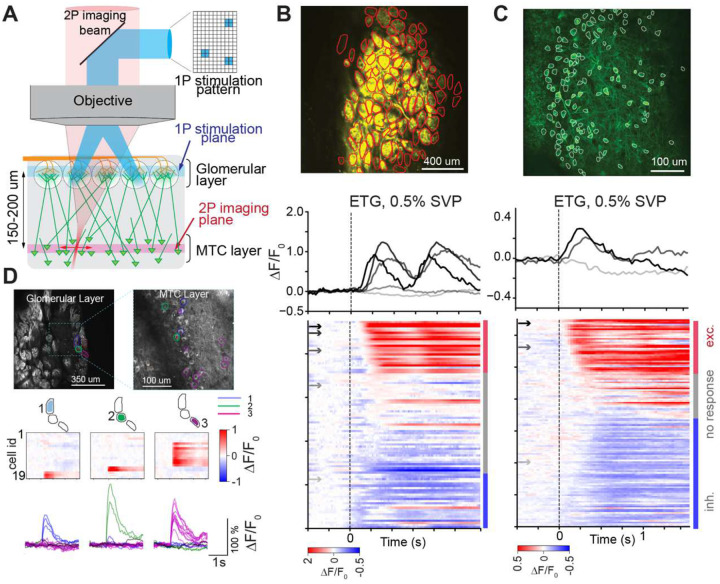
Experimental setup combining a 2P microscope with a 1P DMD-based pattern stimulation system. **A**. The plane of stimulation was focused ~150 μm above the 2P imaging plane, permitting simultaneous stimulation of the glomerular layer and 2P imaging of the MTC layer. ChR2-EYFP was expressed in all OSNs (orange), allowing presynaptic stimulation of individual glomeruli. GCamp6f was expressed in MTCs and their dendrites (green), allowing fast 2P imaging of MTC cell bodies, as well as postsynaptic imaging of glomeruli. **B**. Fast 2P imaging of glomerular activity. *Top:* A field of view containing 95 identified glomeruli (ROIs drawn in red). *Bottom:* Temporal profiles of 5 glomerular odor responses and a heat map of activity across all recorded glomeruli in response to a presentation of ethyl tiglate (ETG) at 0.5% SVP (responses aligned to the onset of the first inhalation following 1s odor presentation)(mean of 5 repetitions for 95 glomeruli in 1 mouse (Tbet-Cre x Ai148).). **C**. Fast 2P imaging of MTC activity. *Top:* A field of view containing 130 MTCs expressing GCaMP6f (Tbet-Cre x Ai148). *Bottom:* Temporal profiles of 3 MTC odor responses and a heat map of all simultaneously recorded MTCs in response to presentation of ETG at 0.5% SVP (mean of 10 repetitions for 130 MTCs in 1 mouse). **D**. Identification of MTCs connected to specific glomeruli in thy1-GCaMP6f x OMP-ChR2. *Top left:* Mean-fluorescence image of the glomerular layer with 3 targeted glomeruli. *Top right:* Mean-fluorescence image of the MTC layer with MTCs identified as connected to one of the 3 targeted glomeruli. *Middle:* Heatmap of 19 MTC responses to brief optogenetic photostimulation of each of the 3 single glomeruli (mean of 10 repetitions, 20 mW/mm^2^ laser power, 10 ms duration, p < 0.05, paired t-test).). *Bottom:* Temporal profiles of 19 MTC responses to individual glomerular stimulation (mean of 10 repetitions/targeted glomerulus). Each cell is assigned to the glomerulus that evoked the strongest response, defined as the stimulation spot with significantly higher activity compared to all other spots (p < 0.05, paired t-test). Cells are colored according to this assigned glomerulus.

To study the transformation of information from glomeruli to the MTCs, we developed an all-optical approach. We used a transgenic mouse model expressing the fast activity indicator GCamp6f in MTCs and ChannelRhodposin2 (ChR2) in the olfactory sensory neurons (OSNs) (cross between OMP-ChR2-EYFP^29^ and Thy1-GcaMP6f (GP.511)^30^. For numbers of animals in each experiment see Table 1. Our all-optical system combines a digital-mirror-device capable of one-photon (1P) pattern stimulation^31,32^ of the bulbar surface with 2P Ca^2+^ imaging the glomerular and MTC layers (Fig. 1A, Fig. S1A). Our custom 2P-microscope is based on the Janelia MIMMS2.0 design, with an extended field of view (~2×2 mm^2^). This allowed us to image almost the entire dorsal surface of the OB and record post-synaptic glomerular activity at 60 Hz (Fig. 1B), as well as a large number of MTCs (>100) at 30 Hz in the same preparation (Fig. 1C). Shifting the focal plane of 1P stimulation ~150 μm above 2P imaging plane let us image the MTC layer (~150 μm deep) while stimulating individual glomeruli at the dorsal surface with ~3–4 μm lateral resolution. Following comparative study between pre- and post-synaptic recording using different pattern of fluorescent indicator expression^33^, we assumed that postsynaptic glomerular activity recorded using Thy1-GCaMP6f line is a good proxy of an input signal to the bulb.

By aligning glomerular and MTC activity to the first inhalation after odor delivery and averaging across trials, we identified their response latencies and ranked them by their activation order (Fig. 1B, C) (see Methods). To find MTCs receiving direct excitatory inputs from individual glomeruli, we imaged MTC activity in response to glomerular optogenetic stimulation (Fig. 1A, D). We first identified a region of interest (ROI) corresponding to an individual glomerulus using 2P imaging, and generated a precise stimulation pattern matching the shape of the glomerular ROI using the DMD based projector system. This approach allowed us to achieve precise stimulation, with much less excitation of adjacent glomeruli compared to a previously used grid-based square stimulation strategy (see Methods and Fig. S2)^27,31,34^. Stimulation of individual glomerular ROIs (pulse duration: 10 ms, intensity: 15–30 mW/mm^2^) evoked rapid and reliable changes in the fluorescence signal of a few MTCs (2–6 cells) in a field of view centered ~150 μm below the glomerular layer (Fig. 1D). These MTCs were considered to be functionally connected to the targeted glomerulus and were classified as ‘daughter MTCs’ (D-MTCs).

Our method of D-MTCs identification may be affected by off-target stimulation, mostly due to the activation of axons of passage crossing above and beside a targeted glomerulus before terminating in more posterior glomeruli (Fig. S2B). To minimize this effect, we matched the stimulation patterns to the shape of individual glomerular ROIs and used minimal stimulation power sufficient to evoke MTC responses (Fig. S2C). A MTC responding to the stimulation of multiple glomeruli was assigned to the glomerulus, which evoked the stronger response (Fig. S2D). If the response amplitude criterion was ambiguous, we assigned the MTC to the more posterior glomerulus (See Methods).

### Stereotypic responses among daughter MTCs.

**Figure 2.**
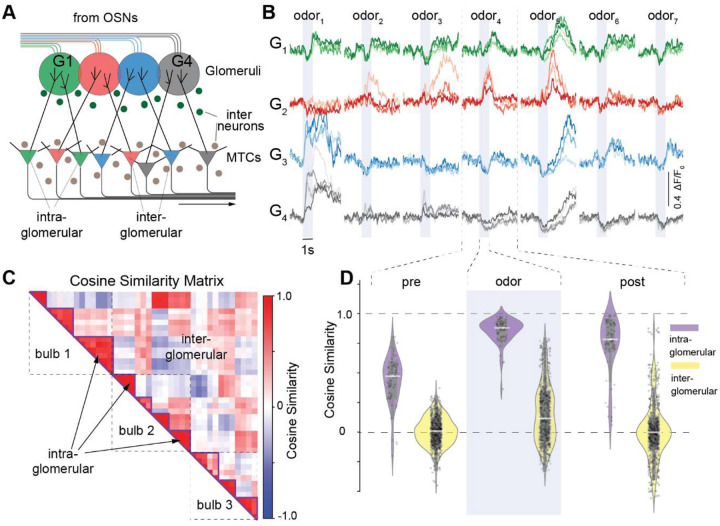
Stereotypy of responses in intra-glomerular channels. **A**. Schematic diagram of the olfactory bulb circuit illustrating the intra- and inter-glomerular connections between glomeruli (colored circles) and MTCs. Glomeruli receive sensory input from olfactory sensory neurons (OSNs) and send processed information to higher cortical areas via the MTCs. Inhibitory interneurons mediate local circuitry between glomeruli and MTCs. **B**. Example traces showing the activity of four identified glomerular channels, as described in Fig. 1D. Glomeruli (G1–G4) are color-coded, and different shades of the same color represent D-MTCs belonging to the same glomerulus. The responses to a sequence of seven distinct odors are shown (odors1-7: 2% heptanoic acid, 0.2% hexanal, 2% hexanal, 0.2% isovaleraldehyde, 2% isovaleraldehyde, 0.2% phenylpropionate, and 2% phenylpropionate). The response periods are divided into pre-odor (2 seconds), odor presentation (1 second), and post-odor (3 seconds) phases (mouse line: OMP-ChR2 x Thy1-GCaMP6f). **C**. Cosine similarity matrix comparing MTC activity across three olfactory bulbs (from two animals, totaling 11 glomerular channels). The matrix displays the response similarity for 12 different odors at 2 concentrations, covering the combined pre-, odor-, and post-odor phases. The matrix is divided into intra-glomerular regions (within the same olfactory bulb) and inter-glomerular regions (across different bulbs). **D**. Violin plots summarizing the distribution of cosine similarity values for intra-glomerular (purple) and inter-glomerular (yellow) MTC pairs during the pre-odor, odor, and post-odor phases (n = 71 intra-glomerular and 749 inter-glomerular pairs in 2 animals, p < 0.001 for each epoch, Mann Whitney U test). Mean cosine similarities were as follows (pre-odor, odor, post odor) intra-glomerular: 0.433, 0.859, 0.745; inter-glomerular: 0.004, 0.152, 0.001. (All presented data collected from mouse line: OMP-ChR2 x Thy1-GCaMP6f).

One open question is the extent to which the output of MTCs is shaped by direct input from their “parent” glomerulus (intra-glomerular signaling) versus lateral signals originating from other glomeruli in the olfactory bulb (inter-glomerular signaling) (Fig. 2A). To examine this, we recorded MTC responses to 12 odors at 2 concentrations in awake animals (n = 3 OBs in 2 animals). We observed that activity patterns were diverse across D-MTCs connected to different glomeruli during odor presentations, while D-MTCs receiving input from the same glomerulus were much more consistent (Fig. 2B, example traces for 14 D-MTCs from 4 glomeruli in 1 mouse, 7 odors, mean of 10 trials). To quantify this similarity, we concatenated activity with 33 ms bins over a 6 s period (time interval from −2 s to 4 s, where 0 is the onset of the first inhalation after odor delivery; odor period is from 0 to 1 s) and measured the cosine similarity between MTC activity vectors within and across glomeruli (Fig. 2C). We observed that cells within the same glomerulus exhibited high similarity (diagonal blocks), indicating that intra-glomerular processing maintains high stereotypy in D-MTC outputs, in contrast to inter-glomerular responses which showed lower similarity (off-diagonal regions). Examining the time course of cosine similarity within each trial epoch (pre-odor: −2 – 0 s, odor: 0 – 1 s, and post-odor: 1 – 4 s epochs) revealed that intra-glomerular D-MTCs exhibited higher stereotypy than inter-glomerular D-MTCs across all odor epochs (n = 71 intra-glomerular and 749 inter-glomerular pairs in 2 animals, p < 0.001 for each epoch, Mann-Whitney U test). Specifically, mean cosine similarities were as follows (pre-odor, odor, post odor) intra-glomerular: 0.433, 0.859, 0.745; inter-glomerular: 0.004, 0.152, 0.001. Notably, intra-glomerular D-MTCs were maximally similar during the odor presentation period (Fig. 2D, Fig. S3). This suggests that processing within each glomerulus leads to highly similar, though not identical, activity in the connected D-MTCs, and this is preserved during odor responses as well as outside of odor response periods.

### The early responding glomeruli and MTCs are preserved across concentrations.

**Figure 3.**
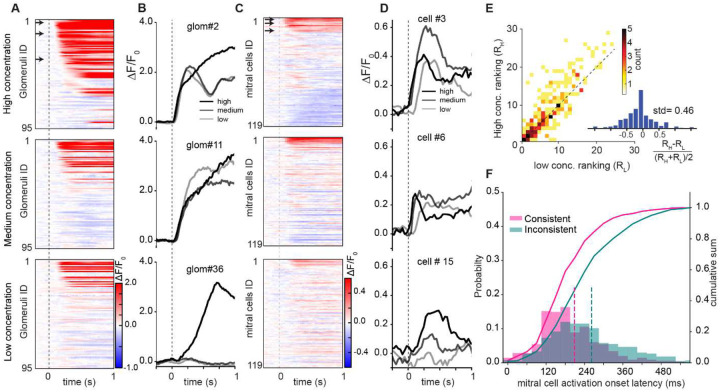
Consistency and timing of odor responses across concentrations. **A**. Odor responses of 95 glomeruli across 3 concentrations (0.1%, 0.5%, and 5% SVP) of ETG ordered by response latency at the highest concentration. **B**. Fluorescence traces for 3 glomeruli marked by arrows in **A**. **C**. Odor responses of 119 MTCs across 3 concentrations (0.05%, 0.5%, and 2.5% SVP) of Acetophenone ordered by response latency at the highest concentration (1 mouse, AAV-syn-FLEX-jGCaMP8f viral injection in Tbet-Cre animal). **D**. Fluorescence traces for 3 MTCs marked by arrows in **C**. **E**. Distribution of glomerular activation ranking in response to low (0.1% SVP) versus high (0.5% SVP) odor concentrations (8 odors, 199 glomeruli, 3 OBs, 230 responding odor-glomerulus pairs, means of 5 repetitions in 3 mice). *Insert*: Distribution of normalized rank discrepancies between high and low concentrations: $\frac{{\text{R}}_{\text{H}}-{\text{R}}_{\text{L}}}{({\text{R}}_{\text{H}}+{\text{R}}_{\text{L}})/2}$. **F**. Distribution of latencies from inhalation for consistent (pink) and inconsistent (green) MTC responses. MTC responses were considered consistent if MTCs had excitatory response to both odor concentrations, and inconsistent if only positively responding to one concentration (two concentrations: 0.25% and 2.5% SVP, 349 consistent cell-odor pairs and 601 inconsistent cell-odor pairs, 7 animals and 8 bulbs, means of 10 repetitions). The mean activation onsets are shown by dashed lines (μ_cons_ = ~190, μ_incons_ = ~250 ms, *p*<1e-4; two-sided Wilcoxon signed-rank test).

To find the concentration invariant features of neural activity, we recorded glomeruli and MTC responses to the same eight odors at two or three precisely controlled concentrations (0.05, 0.2, 2% saturated vapor pressure (SVP)) (Fig. 3A–D). For glomerular recordings (Fig. 3A–B), we ranked the glomeruli based on their activation latencies and found that while the set of responsive glomeruli changed substantially across concentrations, the subset of glomeruli responsive to all concentrations largely retained their rank ordering of latencies (Fig. 3E). We observed that discrepancies between glomerular ranks for high and low concentrations, R_H_ and R_L_, was increased for later activated glomeruli, while the distribution of normalized discrepancies $\frac{{\text{R}}_{\text{H}}-{\text{R}}_{\text{L}}}{({\text{R}}_{\text{H}}+{\text{R}}_{\text{L}})/2}$ remained quite narrow with a large peak at zero (Fig. 3E insert; 8 odors, 199 glomeruli, 3 OBs, 230 significant odor-glomerulus pairs, means of 5 repetitions in 3 mice). The observed changes in glomerular response rankings across concentrations may stem from multiple methodological and biological factors. Glomerular rankings, estimated from onset latency of GCaMP responses, are sensitive to noise, trial variability, and threshold selection, with higher concentrations notably reducing the accuracy of latency calculations. Additionally, the interplay of the temporal slope of concentration raise in the nose with odor inhalation and steepness of dose-response curves of receptor-odor pairs may lead to ranking swaps (Fig. S4).

For MTCs, we found that increasing the concentration of an odorant changed the set of responsive MTCs, but the earliest responses were largely preserved across concentrations (Fig. 3C–D, data shown from MTCs expressing the fast indicator jGCaMP8f). To quantify this effect, we divided responsive MTCs into two groups: consistent, which responded to both high and low concentrations, and inconsistent, responding to only one concentration. The latencies of consistent MTC responses were strongly biased towards the beginning of the sniff cycle, while inconsistent responses generally had longer latencies (consistent: median = 150 ms, n = 349, inconsistent: median = 210 ms, n=601 MTC-odor pairs 8 bulbs in 7 animals, *p* < 1e-4; two-sided Wilcoxon signed-rank test) (Fig. 3F).

These findings provide strong evidence that both early glomerular and MTC responses carry concentration invariant information about odor identity.

### The transformation of odor responses from glomeruli to MTCs.

**Figure 4.**
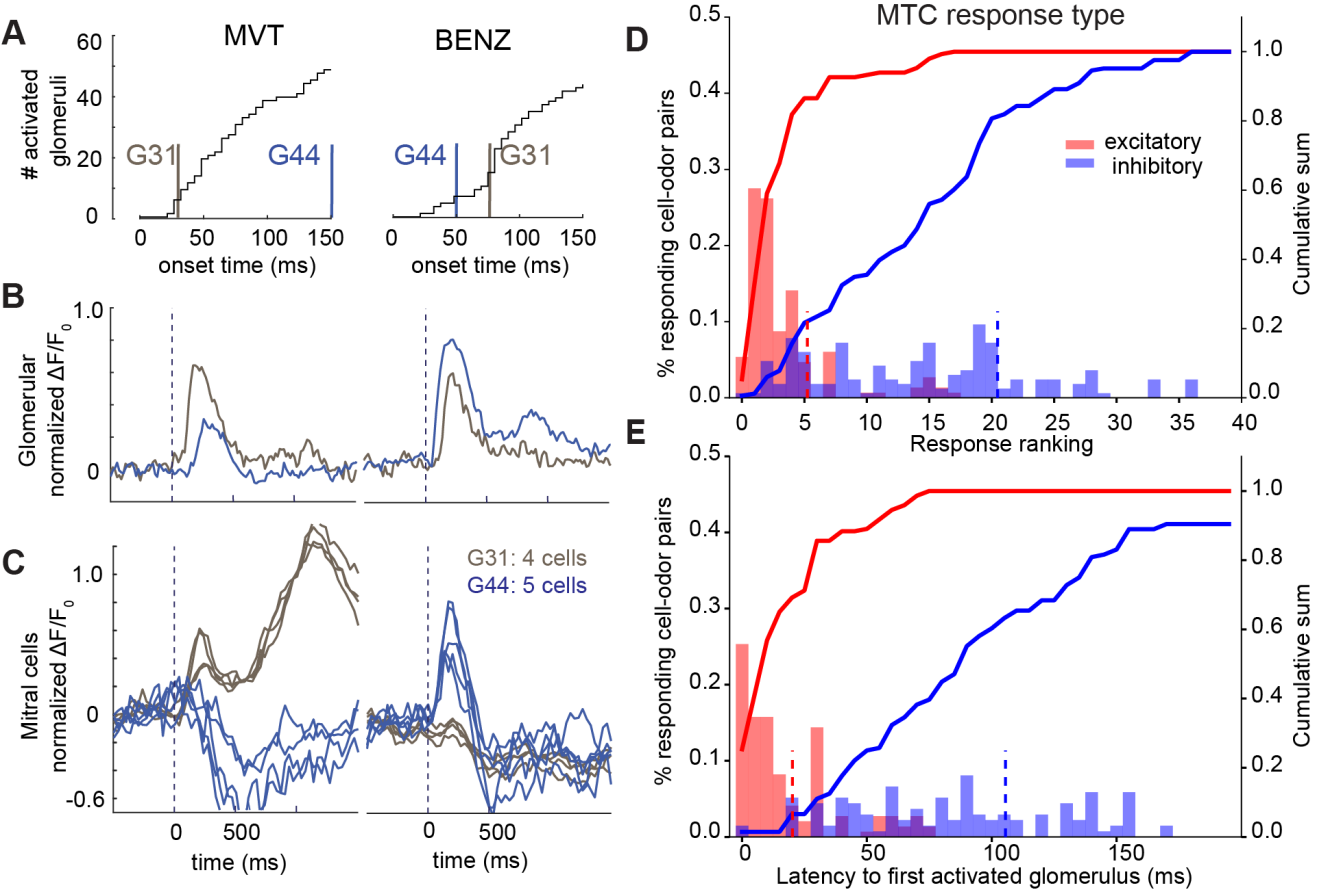
Odor responses of connected P-glomeruli and D-MTCs as a function of latency. **A**. The cumulative distribution of glomerulus response latencies relative to the onset of inhalation for two odors: methyl valerate (MVT, left) and benzaldehyde (BENZ, right). Latencies of two glomeruli (G31-gray and G44-blue) are marked by vertical bars (mean of 5 repetitions in 58 glomeruli). **B**. Mean response of two glomeruli, G31 and G44, to two odors. **C**. Mean responses for D-MTCs of the same glomeruli, G31 (gray, 4 daughter cells) and G44 (blue, 5 daughter cells), to the same odors. **D**. Histogram of response latency rankings for P-glomeruli with excitatory (red) (rank: mean ± std; 5.3±4.8) and inhibitory (blue) (rank: mean ± std; 20.3±11.7). D-MTC responses (9 glomeruli, 32 D-MTCs, mean of 10 repetitions for two OB hemispheres from 1 animal having 58 and 74 recorded glomeruli). The dashed lines indicate the mean of each distribution and solid lines show each cumulative distribution. MTCs having excitatory responses connected to lower rank glomeruli (p<1e-4, one-sided Kolmogorov–Smirnov test) **E**. Histogram of P-glomerular response latencies. The latencies are measured from the onset of the first glomerular response (excitatory responses median latency: 10 ms, 80% range: 30 ms). (All presented data collected from mouse line: OMP-ChR2 x Thy1-GCaMP6f).

As shown in Figs. 1B and 3A, an odor activates a set of glomeruli in a unique temporal sequence^6^, presumably due to the different binding affinity of their corresponding receptors, with high affinity receptors activating earlier after inhalation and low affinity receptors activating later (see Discussion)^35,36^. These glomeruli provide direct excitatory input to their D-MTCs, but it is unknown whether the timing of glomerular activation impacts the nature of responses in their D-MTCs. To address this question, we identified D-MTCs for a subset of glomeruli (Fig. 1D) and analyzed odor responses of MTCs in relation to the response properties of the parent (P) glomeruli. Using 2P imaging, we measured responses of 58 glomeruli to 2 odors (5 repetitions): methyl valerate (MVT) and benzaldehyde (BNZ) (see Methods and Fig. 1B), including two P-glomeruli with identified D-MTCs (G31 and G34). When we ranked the glomeruli by the latency of their responses (Fig. 4A), we found that the rank latencies of G31 and G34 swapped between these two odors. In the Ca^2+^ responses of these two glomeruli and their D-MTCs (Figs. 4B and C) we observed that the D-MTCs of the earlier activated P-glomerulus exhibit strong excitatory responses (G31 for MVT (green) and G44 for BNZ (blue)) while D-MTCs of the later activated glomeruli mostly have inhibitory responses during the early phase of odor delivery, even though the P-glomeruli exhibited a robust excitatory response to the odors.

To explore this phenomenon across many glomeruli-odor pairings, we repeated this experiment for 13 odors and 2 concentrations (0.2% and 2% SVP) for 9 sets of D-MTCs and their P-glomeruli across two OBs in a mouse. Examples for 2 glomeruli are shown in Fig. S5. Across all conditions, we found that D-MTCs connected to P-glomeruli with low ranks (5.3±4.8, mean±std) exhibited excitatory responses, while D-MTCs exhibiting inhibitory responses were connected to P-glomeruli with broadly distributed rankings (20.3±11.7, p<1e-4, one-sided Kolmogorov–Smirnov test test) (Fig. 4D). Response signs were determined based on initial responses after odor delivery. A similar picture emerges for the distribution of P-glomeruli response latencies: D-MTCs with excitatory responses were connected to P-glomeruli that were almost all activated within 20 ms after the first activated glomerulus (median latency: 10 ms, 80% range: 30 ms) (Fig. 4E).

We also observed that, on average, glomerular response amplitude decreased with increased response latency (Fig. S6A, C, E). Thus, we analyzed D-MTCs’ responses as a function of their P-glomeruli’s response amplitude and found a similar relationship as measured for response latency: the D-MTCs connected to P-glomeruli with the highest response amplitude exhibited predominantly excitatory responses (p<1e-4, one-sided Kolmogorov–Smirnov test) (Fig.S6F). To reject the possibility that amplitude-latency dependence was a result of postsynaptic glomerular activation, we tested this relationship in a mouse model where the Ca^2+^ indicator GCaMP6f was expressed presynaptically in the OSNs (OMP-Gcamp6f). Across 3 concentration levels, we observed that higher amplitude responses were correlated with earlier activation onsets, similar to measurements performed post-synaptically (postsynpatically vs presynaptically : low conc. slope=−6.9 log(ΔF/F_0_)/ms, R^2^ = 0.98 vs slope=−5.9e-3, R^2^=0.86; mid conc.: slope =−6.8e-3, R^2^ = 0.97 vs slope=−4.7e-3; R^2^ = 0.82; high conc.: slope= −3.8e-3, R^2^=0.71 vs slope=−5.9e-3, R^2^=0.90, 121 glomeruli from two bulbs).

### Temporal interactions between different glomerular units shape odor representations.

Based on our observations of odor responses in defined glomerular units (P-glomeruli and connected D-MTCs), we set out to test the hypothesis that an initial excitatory drive is followed by recruitment of an inhibitory network mediating glomerulus-glomerulus interactions. This will result in the existence of a temporal window during which the MTCs experience primarily excitation from their P-glomeruli. To specifically test the role of P-glomerular activation timing for defining the amplitude of D-MTC responses, we designed an experiment to probe the excitability of D-MTCs using brief optogenetic stimulation of a P-glomerulus as a function of the latency of the pulse relative to the onset of inhalation. We presented optogenetic pulses on their own, and in the presence of odors which excite broad glomerular activity patterns. We expected that, in the presence of a background odor, D-MTCs would only be effectively activated by pulses presented earlier in the sniff cycle, and for the later pulses, the D-MTC responses would be suppressed by a broad inhibitory network response recruited by the large number of earlier odor-activated glomeruli. Using this optogenetic approach allowed us to clamp the activation amplitude of the targeted glomerulus, and thus selectively examine the role of timing for D-MTC excitability.

**Figure 5.**
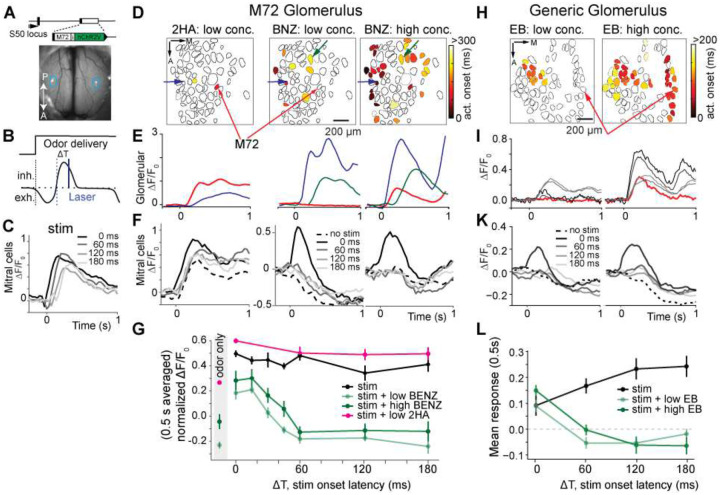
P-glomerulus to D-MTC transmission efficacy as a function of odor and latency. **A**. A single glomerulus was targeted for photostimulation and imaging using the transgenic mouse model M72S50. The genetic sequence encoding the M72 receptor gene and ChR2-EYPF fusion protein was targeted to the M72S50 locus positioned at the center of the dorsal OB. **B**. Timing of odor delivery and photostimulation relative to respiration phase: the odor delivery valve was triggered at the onset of exhalation, so that odor exposure starts at the next inhalation. A laser pulse was delivered at a time interval ΔT after the onset of inhalation. **C**. Mean response of the D-MTCs of the M72 P-glomerulus to optogenetic stimulation timed to different latencies from the onset of inhalation (no odor, 10 ms, ~20 mW/mm^2^, n = 9 D-MTCs, 10 photostimulations). **D**. Odor activation onset latency maps for low concentration 2-hydroxyacetophenone (2HA) (*right*), low concentration benzaldehyde (BNZ) (*middle*), and high concentration BNZ (*left*). The M72 glomerulus is indicated by the red arrow (see Fig S8 for amplitude map). **E**. The response profile of the M72 glomerulus (red) and other glomeruli (blue and green), shown by arrows of the same color in **D**. **F**. Mean response of the M72 glomerulus D-MTCs to odor only (no stim, dashed line) and to an optogenetic pulse delivered at different latencies from the onset of odor inhalation (mean of 5 photostimulations, 10 ms, ~20 mW/mm^2^, n = 11 D-MTCs across 4 mice). **G**. Mean amplitude of D-MTC responses to a light pulse as a function of pulse timing relative to the onset of inhalation. Pulses were delivered without odor (black), with the presentation of 2HA, a strong ligand of M72 receptor (red), and with BNZ, a weak ligand of M72 receptor, at low (light green) and high (dark green) concentrations (mean of 5 photostimulations, 10 ms, ~20 mW/mm^2^, n = 11 D-MTCs across 4 mice, error bars are ±1 SEM, * = p<0.05 paired student’s t-test). (see Fig S7 for individual D-MTC responses) **H**. Odor activation onset latency map for low (*left*) and high concentration (*right*) ethyl butyrate (EB) in an OMP-ChR2 mouse**. I**. The response profile of the target glomerulus (red) and other glomeruli responsive to low concentration EB (colored glomeruli in **H**, left panel). **K**. Mean response amplitude of D-MTCs connected to the target glomerulus for odor only (dashed line) and to optogenetic pulses delivered to the target glomerulus at different pulse latencies (mean of 5 photostimulations, 10 ms, ~20 mW/mm^2^, n = 4 D-MCs for 1 mouse). **L**. Mean amplitude of D-MTCs responses to a light pulse as a function of pulse timing timing relative to the onset of inhalation without odor (black), and in the presence of EB at low (light green) and high (dark green) concentrations. (Additional analyses are provided in Fig S12)

For this experiment, we used a mouse model where the M72 receptor and ChR2 genes were inserted into the S50 receptor gene locus (M72S50-ChR2-EYFP)^32,37^ (Fig. 5A). In this line, the ChR2-EYFP expressing M72 glomerulus was conveniently positioned in the center of the dorsal OB. To image postsynaptic glomerulus and MTC activity, we crossed this line with the Thy1-GCaMP6f mouse line (strain 5.11)^30^.^30^ We then compared the responses of the M72 glomerulus to its strong (2-hydroxyacetophenone, 2HA), and weak (benzaldehyde) ligands^38^ (Fig. 5D–G, Fig. S10B, and Fig. S9). As expected, we observed strong excitatory responses for 2HA in both the M72 P-glomerulus and its connected D-MTCs at both high (data not shown) and low concentrations (Fig. 5E, F; n =11 D-MTCs, 4 mice). For weak ligands such as BNZ, at low concentrations we did not observe activation of the M72 glomerulus, but elicited activation of other glomeruli across the dorsal OB (Fig. 5E–F and Fig. S10B).

When we presented an optogenetic pulse (10 ms, 20–25 mW) without an odor, D-MTC response amplitude was almost independent of the latency of the pulse (11 neurons in 4 mice) (Fig. 5C,G). Pairing pulse presentation with 2HA only slightly increased D-MTC response amplitude. The limited increase of response amplitude was likely due to response saturation. In the presence of low and high concentrations of BNZ, D-MTC responsiveness to light stimulation of the M72 P-glomerulus significantly decreased after a brief interval, ~30 ms (Fig. 5G). This could be explained by inhibition of the M72 D-MTCs from other glomeruli activated by BNZ. This suppression lasted for more than 180 ms, which is consistent with the time course of lateral inhibition measured in *in vitro* studies^39,40^.

Is the result observed for the M72 glomerulus generalizable to other glomeruli? To address this question, we used the OMP-ChR2-EYFP x Thy1-GCaMP6f mouse line introduced previously. As above, we identified the D-MTCs of a specific glomerulus and performed odor imaging in the glomerular layer using a battery of odors at different concentrations (Fig. 5H). We identified 5 glomeruli and ligand pairs (n=5 mice), so that 1) the chosen ligand activated the glomerulus only at a high concentration, and 2) we could find D-MTCs for that specific glomerulus in the field-of-view. For example, one such glomerulus responded to ethyl butyrate (EB) at high concentrations, but not at low concentrations (Fig. 5I). A similar response pattern was observed for ETG (data not shown). Additionally, its response latency to high concentrations of EB was delayed compared to other glomeruli. Thus, EB could be considered a weak ligand of the target glomerulus (Fig. 5I, K). We investigated the responsiveness of its D-MTCs to an optogenetic pulse (10 ms, ~20 mW/mm^2^) with and without presentation of EB. Like the M72 glomerulus, the responsiveness of D-MTCs significantly decreased for light pulses presented at 60 ms or later after the onset of inhalation. (Fig. 5K, L). These findings strongly support the existence of a time-dependent filter, putatively mediated by glomerulus-glomerulus inhibitory interactions, as a general computational principle in the OB network. The excitatory drive from early activated glomeruli precedes the inhibitory drive, thus creating a brief temporal window during which MTCs experience excitation from their parent glomerulus and a lack of inhibition from other glomeruli.

**Figure 6.**
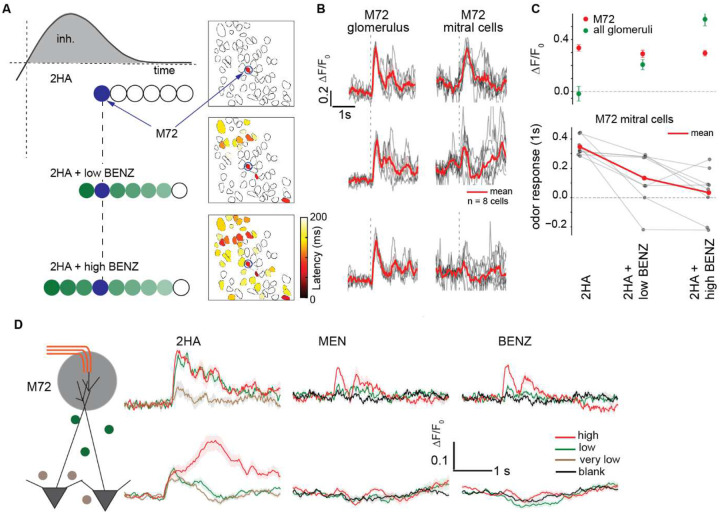
M72 glomerulus odor response transmission in the presence of weak and strong ligands. **A**. *Right:* Mean glomerular activation latency in response to the presentation of 2HA at low concentration (100 times air diluted 2uL in 5 ml mineral oil solution) (*top*), a mixture of 2HA and a low concentration of benzaldehyde (BENZ) (0.2% SVP) (*middle*), and a mixture of 2HA with a high concentration of BENZ (2% SVP) (*bottom*) (n = 59 glomeruli in 1 mouse, mean of 5 repetitions, inter-trial interval of 60s). The M72 glomerulus is indicated by the blue arrow and circle. *Left*: Schematics of the timing of activation across different glomeruli in response to these stimuli. **B**. Mean fluorescence of the M72 glomerulus (left), and its connected D-MTCs (right) for the same odor conditions as in **A** (error shading is ±1 SEM, mean of 5 repetitions (grey lines), n = 1 glomeruli and 8 MTCs, dotted line is onset of inhalation). **C**. The M72 glomerulus and mitral cell odor response for different odor conditions: 2HA alone, 2HA with low BENZ, and 2HA with high BENZ. The red line represents the mean response across 8 cells, with individual cell responses shown in gray. Glomerulus signal doesn’t change adding BENZ to 2HA (t-statistic <1.4 with a p-value>0.05, paired-t test). M72 D-MTC (8 cells from 2 mice) responses were suppressed with a t-statistic=3.17 and p-value<0.008 for 2HA + low BENZ; with a t-statistic=4.08 and p-value<0.003 for 2HA + high BENZ, paired t-test). **D**. Glomerular and D-MTC responses to multiple concentrations of the strong ligand 2HA and weak ligands (BENZ and Menthone [MEN]). While weak glomerular responses to 2HA were sufficient to drive robust D-MTC activation, stronger glomerular activation by weak ligands (BENZ and MEN) failed to elicit significant D-MTC responses. (All presented data collected from mouse line: M72S50 x Thy1-GCaMP6f).

Having established and characterized the excitatory temporal window using our optogenetic approach, we designed an experiment to further test the validity of this phenomenon using odorant stimuli. We observed that the presentation of 2HA at low concentration (2 μL 2HA in 5 mL mineral oil, 100 times diluted in air) activated only the M72 glomerulus in the field of view, and evoked robust activation of all M72 D-MTCs (Fig. 6 A left panel, B top panel). Next, we presented a mixture of 2HA and BNZ, which activated additional glomeruli besides M72: depending on the relative concentration of 2HA and BNZ, the most sensitive glomerulus for BNZ was activated either before or after activation of M72. When we mixed 2HA with BNZ at low concentration, D-MTC responses decreased only slightly, presumably because activation of the M72 glomerulus by 2HA preceded activation of the majority of the glomeruli recruited by BNZ (Fig. 6A middle, and B – second row). However, when we increased the concentration of BNZ, BNZ evoked responses in multiple glomeruli earlier than the response of M72 (M72 glomerulus signal doesn’t change with adding BENZ to 2HA, mean of Ca^2+^ fluorescence amplitude over 0.5 s following odor inhalation onset, t-statistic <1.4 with a p-value>0.1, paired-t test), and the M72 D-MTC responses were suppressed (Fig. 6A – right, B- third row with a t-statistic=3.17 and p-value<0.008 for 2HA + low BENZ; with a t-statistic=4.08 and p-value<0.003 for 2HA + high BENZ, paired t-test; 8 D-MTCs in 2 mice.). This result further supports our conclusions from the optogenetic probing experiments, that the MTCs of later activated glomeruli are suppressed by earlier activated glomeruli. To further investigate the influence of other glomeruli on D-MTC responses, we tested multiple odor concentrations of strong (2HA) and weak ligands (BNZ and Menthone [MEN]). While weak glomerular responses to 2HA were sufficient to drive robust D-MTC activation, stronger activation of glomeruli by weak ligands (BNZ and MEN) failed to elicit significant D-MTC responses (Fig. 6D).

## Discussion

Prior to being sent to the cortex, odor information from individual receptor neurons is integrated in the glomeruli^41^, and then transformed and reformatted into the activity of MTCs. To understand this transformation, we focused on the processing performed within a single glomerular unit in awake and freely breathing animals. Employing an optogenetic targeting strategy, we identified MTCs connected to specific glomeruli, and measured odor and light evoked responses in both P-glomeruli and their corresponding D-MTCs. We found that earlier, but not later responses were preserved across concentrations of the same odor. Moreover, MTCs linked to early activated glomeruli displayed stereotypic excitatory responses, while those connected to later activated glomeruli exhibited variable and diverse responses. To understand this phenomenon, we probed MTC responsiveness to pulsed glomerular optogenetic activation in the presence of odors. We found that MTCs could effectively transmit a glomerular signal to the cortex only in a short temporal window at the beginning of the sniff cycle. Beyond this window, the number of MTCs responding to excitatory input from their P-glomeruli was limited, due to odor-evoked lateral inhibition from early-responding glomeruli. Furthermore, by conducting analogous odor-mixture based experiments we confirmed that odor processing is governed by the same rules. These observations were enabled by technological advances that allowed us to monitor and manipulate networks at relevant spatial and temporal scales. We developed a novel optical system, which enables surface patterned optogenetic stimulation with simultaneous fast 2P Ca^2+^ imaging of deeper layers. We employed an odor delivery system which allowed the presentation of a diverse array of odor stimuli spanning an 8,000-fold concentration range, precisely controlled for temporal accuracy using a closed loop system synchronized to the animal’s breathing (Fig. S14). These tools permitted identification of glomeruli and their functionally connected MTCs, and imaging their responses to odor and optogenetic stimuli.

### What are the underlying neural mechanisms of temporal filtering?

**Figure 7.**
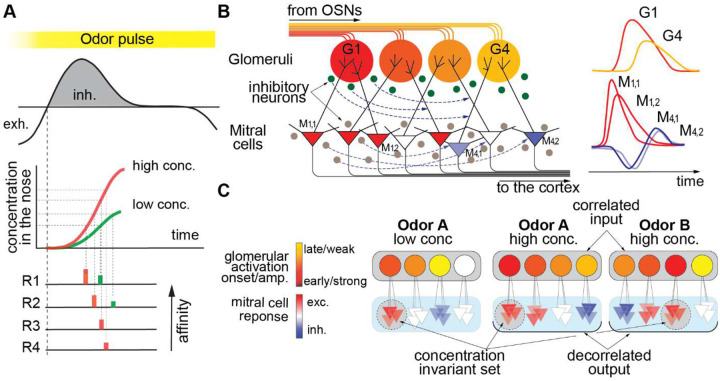
A temporal filtering mechanism responsible for concentration invariant odor identification and odor decorrelation. **A**. Schematics of a respiration cycle (*top panel*) and the temporal profiles of odor concentration in the nose for low (green) and high (red) concentrations (*middle panel*). Schematics of the temporal sequences of receptor activation for low and high odor concentrations (*bottom panel*). Individual receptors become activated when the concentration in the nose crosses a specific threshold. The sequence of activation for the most sensitive receptors remains the same for low and high concentrations of the same odor. **B**. The processing of odor information in the olfactory bulb network. Glomeruli (colored circles) receive input from the OSNs. The glomerulus color indicates the latency of activation in the respiration cycle for an odor. The MTCs (triangles) receive their excitatory inputs from glomeruli and are modulated by inhibitory neurons (small circles). The D-MTCs of an early glomerulus (red color) receives mainly excitatory input without inhibitory modulation. The D-MTCs of later glomeruli (orange and yellow) have mixed responses, being excited by their parent glomeruli as well as inhibited by previously activated inhibitory neurons. *Right panel:* the schematics of glomeruli and MTCs responses. There are two large classes of inhibitory neurons in the OB: Periglomerular cells (green circles), which receive their input from the glomeruli, and granule cells (brown circles), which receive their inputs from MTCs. **C**. Odor coding at the glomerular and MTC levels. For low and high concentration of odor A, the sequence of glomerular activation (indicated by color) is preserved. At the MTC level, the MTCs that receive input from early activated glomeruli (red) have similar excitatory responses for both high and low concentrations and are thus concentration invariant. Two odors A and B have a similar spatial pattern of glomerular activation, but a different sequence of activation. In this case temporal filtering leads to very different patterns of activity at the MTC level, i.e., the odor responses become decorrelated.

Odors evoke sequential activation of multiple glomeruli (Fig. 3A), and we found that the early portion of these sequences were mostly preserved across different concentrations (Fig. 3E). One of the potential mechanisms for such sequential activation is that odor dynamics within the nose lead to a rising instantaneous odor concentration at the receptor epithelium across the inhalation period and results in affinity-dependent receptor activation latencies – where more sensitive receptors are activated earlier (Fig. 7A). While simplistic, this biophysical picture provides a plausible framework for understanding the observed sequential glomerular activation, which plays a crucial role in shaping the dynamics at the next level of processing. The earlier activated glomeruli evoke excitatory responses in their D-MTCs. Concurrently, they drive inhibitory cells in the OB network, which in turn initiate a delayed inhibitory drive. The difference in latency between the inhibitory and excitatory drives opens a brief temporal window when glomeruli can excite their connected MTCs, which we demonstrated with optogenetic pulse probing experiments (Fig. 5).

Which specific cells could shape the inhibition-mediated temporal filtering of MTC responses? The OB’s lateral inhibition is mediated by a diverse array of neurons, both at the glomerular and mitral cell layers. Within the glomerular layer, the short axon cells are capable of inhibiting external tufted cells in glomeruli located at distances up to millimeters away^39,40,42,43^. Since external tufted cells are responsible for initiating excitation across the glomerulus, inhibiting them would naturally result in the suppression of odor-driven spiking in mitral cells^44,45^. In the mitral cell layer, the interaction between MTCs is facilitated by parvalbumin interneurons (PV) and granule cells (GC). PV interneurons exhibit an expansive spatial inhibition profile, thereby imparting widespread modulatory influence across the mitral cell layer, while GCs exert a more focal impact^20,46^. The observed similarity of the inhibitory responses across the D-MTCs of later activated glomeruli (Fig. 4C and S5) may point to a shared source of inhibitory drive acting on their P-glomeruli and thus potentially governed by the short axon cells of the glomerular layer. We compared results of pre-synaptic (Fig. S13) and post-synaptic^47–49^ glomerular optogenetic stimulation. Stimulating MTCs dendrites (using mice expressing ChR2 in MTCs), Arenkiel et al (2007)^47^ reported that an increase of the size of the stimulation spot from 100 to 300 μm diameter does not change the number or firing rate of MTCs [^47^, Fig. 6], while Bolding and Franks (2018)^49^ using the same mouse model observed that widefield stimulation of the OB surface evoked long lasting MTC responses. In these experiments, the optogenetics stimulation bypassed intraglomerular inhibitory network, and directly activated MTCs. In contrary, in our paradigm, we stimulated axons of OSNs in glomerular layer and observed a strong MTC response suppression with an increase of the size of stimulated area, which can be explained by intrabulbar inhibitory network activity, potentially mediated by short-axon cells^39,40,42,43^. However, we cannot rule out structured inhibition of mitral cells via GCs and PV interneurons. The former mechanism, which requires a more detailed investigation, is thought to play a role in shaping MTC responses in behaving animals^38,50–53^.

### What is the functional significance of the temporal filtering?

A tight temporal window, when MTCs are receiving mostly excitatory inputs from their P-glomeruli, may confer multiple computational benefits for odor processing. First, it can potentially implement primacy coding-based concentration invariant odor recognition^27,54,55^ According to the primacy model the earliest activated glomeruli are responsible for encoding an odor’s identity, and consistent early activation of the most sensitive glomeruli at low and high concentration of the same odors as shown in our recordings (Fig. 2A,B&E) provides the conditions for concentration invariant odor encoding. Remarkably, the consistency across concentrations for the set of MTCs activated in a brief temporal window early in the inhalation phase (Fig. 2C,D&F) enables the propagation of concentration invariant odor identity information to higher brain areas. In contrast, the diverse MTC odor responses later in the sniff cycle vary strongly with concentration, and thus are likely to encode complex olfactory attributes rather than invariant odor identity information.

Where is the concentration invariant odor representation formed? Our results highlight the possibility of temporal filtering via delayed inhibition within the OB as a potential neural mechanism (Fig. 7). Previously, Bolding & Franks observed a temporal filtering mechanism implemented in the piriform cortex^49^, where the earlier activity of piriform cortical neurons was concentration invariant, and recurrent inhibition provided a mechanism for filtering out other signals. Our observations complement these findings, and suggest that concentration invariant odor representation actually starts forming in the OB - cortex receives an already pre-processed signal and amplifies the effect initiated in the OB.

Temporal filtering may play another role in odor information processing. Olfactory receptor neurons and their corresponding glomeruli usually respond to many odors, and the responses to different odors may be quite overlapping, especially for chemically similar odors. The amplification of earlier responses and partial suppression of later responses at the next stage of processing may be responsible for decorrelating the signals from different odors (Fig. 7C). See also Fig. S7 for analysis of decorrelation of the signals in transformation from glomeruli to MTCs. The temporal structure of the observed inhibitory drive effectively amplifies earlier signals and suppresses later responses, thus increasing the contrast between spatially overlapping, but temporally distinct, odor evoked glomerular patterns. Decorrelation due to temporal filtering occurs on a rapid time scale, exerting an influence immediately following an initial temporal window of 30–40 ms, which is much faster than previously proposed timescales of decorrelation^18^. Such a neural computation is compatible with behavioral results demonstrating rapid decisions about odor identity^56^ and masking experiments limiting the temporal window of odor information available for odor judgements to <100 ms^28^. Further, temporal filtering can help explain how rodents are able to discriminate between different ratios of a binary odor mixture and generalize this discrimination criterion across a range of concentrations^57^.

### Amplitude vs Timing:

In sniffing animals, the amplitude and activation onset timing are tightly coupled at the olfactory bulb (OB) input level, as evidenced by our data (e.g., Fig. S6A, C, E). Figures S6F, 7, and 8 further confirm the equal significance of amplitude-based ranking in this context. Given the pervasiveness of this coupling, the functional relevance of decoupling amplitude and timing remains elusive, even if response rate were considered more critical, as timing offers robust explanatory power, particularly in reaction time dynamics. Nonetheless, our study has generated several observations that provide valuable insights into this complex and multifaceted issue, needs further investigation.

### Limitations of optogenetic approach.

Our conclusions are based on applying a new ‘functional optogenetic connectivity’ strategy to establish glomerulus-MTC connectivity. The stereotypy of the MTCs responses (Fig. 2) support the robustness of this assignment method, but nevertheless the results may differ from anatomical connectivity in two ways. First, some MTCs may respond *indirectly* to glomerular light activation without being directly connected to the targeted glomerulus (for false positive due to axonal passage stimulation; see Methods and Fig. S2 for details on mitigation strategies). From a computational perspective, these ‘false positive’ MTCs are excited by the p-glomerulus and participate in the same computation, and it may not matter if their connection is indirect. Second, we may miss some connected MTCs. However, in practice we have never seen such ‘false negative’ MTCs which were not responsive to glomerular light stimulation but had excitatory responses to the p-glomerulus odor activation. Overall, these potential discrepancies will thus not affect the study’s conclusions.

### Limitations of Postsynaptic Latency Measurements as Proxies for OSN Activity Onset:

Our conclusion regarding transformation within the olfactory bulb is based on postsynaptic glomerular imaging. However, the postsynaptic glomerular signal comprises contributions from various cell types, influenced by differences in genetic labeling. Specifically, we chose the Thy1-GCaMP6f line, which predominantly labels external tufted cells (eTCs) that closely follow OSN activity, as opposed to the Tbet-Cre labeling, which targets a broader range of excitatory cells, including primarily MTCs. Prior study^33^ have demonstrated that periglomerular cells and eTCs closely follow presynaptic OSN activity, while MTCs exhibit slightly longer latencies than OSNs. Overall, our latency measurements likely represent an upper boundary of presynaptic latencies, and this limitation does not alter the study’s conclusions

### Broad context and open questions.

One of our interesting observations is that MTC activity is not a simple copy of glomerular activation. The responses of MTCs are not fully defined by their apical dendritic signal, but can be modulated by the inhibitory network^58^. This phenomenon has been characterized in zebrafish^59,60^ and flies^61–63^, but has not been explored in the mammalian system where it can be connected to temporal neural dynamics and linked to behavior.

While our results generalized across the output neurons in the olfactory bulb (OB), specifically mitral cells (MCs) and tufted cells (TCs), the majority of our recordings were comprised of cells in the mitral cell layer. These two types of neurons differ in their odor evoked activity^52,64,65^ and projection targets^66^. In our recordings, we observed TCs tended to follow their glomerular input more closely compared to MCs (Fig. S8), resulting in less temporal filtering overall (Fig. S12).

Our findings resolve previously ambiguous observations regarding the diversity and stereotypy of odor evoked activity across intraglomerular D-MTCs, as well as clarify the relationship between odor evoked responses of parent glomeruli and their D-MTCs. For the first time in awake mice, we compared odor responses at both the input glomerulus and multiple D-MTCs simultaneously. This revealed that while D-MTCs were highly consistent with each other in their sign and magnitude of response to different odorants (Fig. 2), they could vary dramatically from the response of their parent glomeruli (Fig. 4 and S5). Furthermore, we found that the odor response similarity between a parent glomerulus and its connected D-MTCs was dependent on the timing of the response of the parent glomerulus. This followed a general rule, whereby, for a given odor, early responding glomeruli passed their activation to their connected D-MTCs, while later responding glomeruli did not consistently do so, a novel mechanism here described as *temporal filtering*.

Our model (Fig. 7) synthesizes these findings and settles ongoing controversy about the nature of the interactions between parent glomeruli and their connected D-MTCs. Previous foundational results in anesthetized mice showed either high stereotypy^31^, or high diversity^67,68^ in the rate of odor responses between intraglomerular D-MTCs. These studies proposed that D-MTCs either 1) follow the response magnitude of their input glomerulus closely, but differ from each other in their precise response phase^31^, or 2) differ greatly in their odor response rate, both from their input glomerulus and each other^67^. While very intriguing, odor responses and neural dynamics are significantly different between awake and anesthetized animals^69,70^, leaving it unclear to what degree these findings could relate to the awake olfactory bulb and how much of the discrepancy between these studies could be accounted for by anesthesia. In a subsequent study focusing on a single glomerular channel in awake mice, Arneodo et al. established that a high affinity ligand of the M72 glomerulus evoked strong and excitatory D-MTC responses, while medium and weak ligands evoked responses that were diverse across D-MTCs^38^. Critically, our results differ from Arneodo et al.^38^ as in their study M72 D-MTC odor responses were recorded in different animals and later pooled, which likely accounts for the diversity among M72 D-MTCs for non-high affinity ligands. This leads to a potential explanation that while M72 D-MTCs may share tuning within each animal, they may differ in their tuning to low affinity ligands across mice. Furthermore, this study supports a new model, that in awake mice the timing of glomerular responses determines the ability of a parent glomerulus to drive its connected D-MTCs, and that intraglomerular D-MTCs are highly stereotyped relative to each other, but not always to their parent glomerulus.

Here our observations lead us to propose that two significant functions of the signal transformation from glomeruli to MTCs are 1) for shaping concentration invariant odor representations, and 2) for rapid decorrelation of glomerular patterns.

## METHODS

### Animals.

In all imaging and stimulation experiments we used adult mice, aged 8–10 weeks at the start of the experiment, comprising both females and males. These mice expressed GCamp6f in mitral and tufted cells (MTCs) and ChannelRhodopsin2 (ChR2) in olfactory sensory neurons (OSNs). This expression was achieved through a cross between OMP-ChR2-EYFP^29^ and Thy1-GCaMP6f (Line #5.11)^30^ mouse lines.^30^ For a specific subset of experiments targeting the M72 glomerulus, we employed adult homozygous M72-IRES-ChR2-YFP mice (strain Olfr160tm1.1 (COP4*/EYFP)Tboz. Additionally, we used Ai148 (Strain #: 030328) X Tbet-Cre (Strain #: 02450) animals instead of GP5.11 for some experiments. For OSN imaging we used the GCaMP6f reporter line Ai93D (JAX Strain #:024103) crossed with OMP-Cre (JAX Strain #:006668). The animals were housed in a controlled environment with a 12-hour reverse light/dark cycle (lights on at 20:00 h). Animals were group housed until surgical implantation, and then kept in isolated cages in a temperature and humidity-controlled animal facility. All animal care and experimental procedures strictly adhered to protocols approved by the New York University Langone Medical Center Institutional Animal Care and Use Committees.

The following animals were used in specific experiments:

### Surgical preparation.

Mice were anesthetized with isoflurane during surgical implantation (2.0% during induction, 1.5% during surgery). A circular craniotomy (~ 3mm diameter) was performed to expose both hemispheres of the dorsal olfactory bulb, extending from the rostral rhinal vein to the naso-frontal suture, centered on the midline, using an air-driven dental drill (Midwest Tradition, FG 1/8 drill bit). A cranial window, a glass 3 mm diameter coverslip, was implanted and secured using a combination of self-curing resin (Orthojet, Lang Dental) and cyanoacrylate glue (Krazy Glue). In addition, a custom 3D-printed diamond-shape headpost^71^ was securely affixed to the skull using C&B Metabond dental cement (Parkell). To ensure adequate recovery, each animal was allowed a minimum of 10 days before the start of experiments.

### Imaging and photostimulation.

For all-optical imaging and photostimulation experiments we built a two-photon (2P) microscope based on the MIMMS 2.0 design (HHMI Janelia Research Campus, Ashburn, VA) and added an arm for patterned photostimulation (Fig. S1A). 2P fluorescence of GCaMP6f was excited at 930 nm using a mode-locked, pulsed Ti:Sapphire laser with an 80 MHz repetition rate, ~120 femtosecond pulse duration, and dispersion compensation (InSightX3, SpectraPhysics, Mountain View, CA). The beam was relayed and magnified by a telescope (scan lens, f = 50 mm, and tube lens, f = 200 mm) to the back-aperture of a 16x/0.8-NA water immersion objective lens (Nikon). Images were acquired at 30 Hz using resonant-galvanometer raster scanning (Cambridge Research) for MTC somata and 60 Hz for the glomerular layer. Emitted photons were reflected by a dichroic mirror (zt575/140dcrb-uf1 custom laser bandpass reflective dichroic, Chroma) and separated to either green or red channels via a dichroic mirror (DM2, 565dcxr, Chroma) and fluorescence was detected using GaAsP photomultiplier tubes (H10770PB-40, Hamamatsu). Images were digitized and recorded using ScanImage 2019 software^72^ (Vidrio Technologies). The stability of the imaging and head-fixation system permitted imaging of the same FOV over multiple sessions. Estimated average power at the objective front aperture ranged from 50 – 100 mW.

1P photostimulation was performed using a digital micromirror device (DMD) projector system (ALP-4.3, Vialux, Germany). The output (timing and power) of a 473 nm diode-pumped solid-state laser (CNI Laser MBL-N-473, 1.5W) was controlled by an acousto-optic modulator (AA Opto-Electronic, MTS110-A3-VIS) and coupled to an optical fiber via a coupler (PAF-X-11-PC-A, Thorlabs). The fiber output was first passed through a piezo-driven homogenizer (Mightex) to remove laser speckle, then collimated, expanded (~10 mm in diameter) and projected to the DMD. The DMD-generated light patterns were relayed and magnified by two additional telescopes, 3:2 (75 mm and 50 mm) and 5:3 (100 mm and 60 mm) and was combined with the imaging path using a dichroic mirror (MGP01-350-700, Semrock) at the conjugate image plane. 1P stimulation patterns were projected ~150 μm shallower than the 2P imaging plane, which focused the patterns onto the glomerular layer imaging in the MTC layer. To compensate for the variable distance between MTCs and the glomerular layer, the 1P stimulation beam was designed with a lower numerical aperture (NA), so that the lateral resolution of the light excitation patterns remained stable along the axial-direction for ~100 μm (Fig. S1B&C).

The point spread function (PSF) of the 1P photostimulation was characterized using a widefield microscope (Fig. S1A–B–C). A thin (< 5 μm) fluorescent layer was illuminated with a pattern, and the excited fluorescence signal was collected through the objective lens, the tube lens and a mirror (MGP01-350-700, Semrock) onto a CMOS camera (Allied vision Prosilica GT1920). The axial dimension of the photostimulation was measured by an inverted scope configuration. Full width at half maximum calculations for the lateral (x,y) and axial (x,z) dimensions were performed after correcting for a slight tilt (10^0^) along the (x,z) plane.

The DMD allowed for generation of spatiotemporal patterns at the sample plane with <3 μm spatial and <1 ms temporal resolution. The axial spread of the beam was approximately 100 μm for patterns of glomerular size. This approach ensured the activation of glomeruli at various depths, whose position could vary due to a nonzero angle between the head-fixation clamp and the cranial window (Fig. S1D).

### Odor delivery.

A three-cassette air-dilution olfactometer was used to prepare and deliver odors with specific concentrations (slight modification from Nakayama & Rinberg 2022). Total clean airflow was maintained at 1L/min by a mass flow controller (MFC) (Alicat MC-1SLPM-D/5M/5IN). Each olfactometer cassette consisted of an MFC for each odor line (0–100 mL/min, Alicat, MC-100SCCMD/5M/5IN), two inline Teflon four-valve manifolds, (NReserach, 225T082), one on-off three-port bypass valve (NResearch, TI1403270), and eight odor vials. Odors were prepared as neat or diluted in mineral oil and stored in amber volatile organic analysis vials (Restek, 21797). While all odor valves were closed, air from the carrier MFC and the odor line MFC of each cassette flowed through a bypass valve, and the combined flow equaled a total of 1,000 mL/min. This airflow was passed through a ‘final valve’ (two 3-way valve, NResearch, SH360T042) to an exhaust line. Clean air was delivered through the final valve to the odor port via a separate clean air line controlled by another MFC at ~1000 mL/min. The flow at the entrance of the nose port is reduced to around 500 mL/min by vacuuming half of it. To deliver an odor, the bypass valve on one of the cassettes was closed while simultaneously a pair of odor valves was opened. Air flow passed through the vial headspace and merged with the clean air line, thus providing air dilution the odorized headspace. To present a mixture of odors, both bypass valves were closed and two pairs of odor vials from two cassettes were opened simultaneously. Concentrations of an individual odor, or components of an odor mixture, were controlled by changing the flow rate of the MFCs in the odor line. For example, to deliver a high concentration of an odor, an odor-line MFC was set at 100 mL/min. After flow was stabilized (at least 2 s), the final valve was switched from clean air flow to the odorized flow, and odorized air was delivered to the odor port with a <40 ms latency. At the end of stimulus presentation, the final valve switched back to delivering clean air to the odor port. As liquid dilution ratios do not accurately predict the headspace (gas-phase) concentration for many odors^73^, liquid dilutions were assayed using a photo ionization detector (Aurora Scientific) to determine relative concentrations of the odorant in gas-phase. For concentration-series experiments, all dilutions were made in the air-phase by diluting odorized air with clean air using a serial diluter, a combination of two MFCs^28^.^28^ For instance, to achieve a 10-fold reduction in the concentration of an odor, the flow rates through both a vacuum MFC and an air MFC were adjusted to 900 mL/min. (https://github.com/olfa-lab/olfactometry/tree/master/device_firmware/serial_repeater_teensy).

All odors are obtained from Sigma-Aldrich and 0.5–1 mL of each odor was placed in a 45 mL vial, except 2HA, which was prepared as a liquid dilution (2uL in 5 mL mineral oil) to reach lower concentration. The following odors were used in all experiments:

### Closed loop system.

Timing of photostimulation delivery was controlled in a closed-loop manner relative to sniff phase. Respiration was monitored using a pressure transducer coupled to a custom Teflon “odor port,” which continuously passed filtered air over the mouse’s nostrils at a rate of 0.5 L/min^54^. Pressure change relative to atmospheric pressure was measured using an externally mounted pressure sensor (24PCEFJ6G, Honeywell) positioned in front of the animal’s nares. This pressure signal was amplified (AD620, Analog Devices), and a Schmitt (dual-threshold) trigger was used to define inhalation and exhalation onsets in real-time on an Arduino microcontroller. Once a trial was initiated, after flow was stabilized (2 s), the final valve was switched by exhalation onset and odor was delivered to the odor port. The system then detected the first inhalation onset and delivered photostimulation at a variable ΔT delay after inhalation onset by triggering a TTL pulse from the pulse generator. This TTL pulse was used to time three additional TTL pulses from a stimulator: PMT gating, DMD frame trigger, and AOM on-state. In most of our experiments, the DMD frame trigger and AOM on-state pulse durations were set to 10 ms. The PMT gating signal started 2 ms earlier and ended 2 ms later to ensure gating.

### Online motion correction.

To facilitate imaging the same field of view within and across days, we implemented a previously described online motion correction method^74^. The FOV was first aligned to a reference image manually, then the position was fine-tuned automatically using a custom designed closed-loop algorithm, implemented as a module within the ScanImage software package^72^ (Vidrio Technologies). This algorithm attempted to minimize the difference between the reference image and the FOV by iteratively moving the microscope stage (Thorlabs PLS-XY) to reduce the residual displacement computed using a rigid motion correction package (NoRMCorre, Flatiron Institute^75^. The optimization typically converged within 10–15s once the residual displacement vector was reduced to < 0.5 mm in magnitude. Apart from aligning the FOV across multiple days, we also performed this motion correction routine between consecutive blocks of imaging. This allowed us to maintain precise alignment and compensate for any potential drift in the FOV during the experiment.

### Segmentation of glomeruli.

ROIs were manually selected based on mean or maximum intensity projections. However, this method was insufficient to detect overlapping glomeruli. Furthermore, glomeruli can vary in size and shape making it unreliable to solely depend on maximal fluorescence projections. Therefore, following the method described in^76^, we obtained a low-dimensional representation of odor responses across glomeruli. First, we computed the singular value decomposition (SVD) of each trial,*F*_*i*_ (10 sec duration) and kept the top 20 eigenvectors. We then calculated the spatial projections of each trial onto these components, *U*_*i*_ = *F*_*i*_*V*_*i*_. Each matrix *U*_*i*_ consisted of the left singular vectors *F*_*i*_, scaled by the singular values and was therefore a 20-dimensional summary of the segment *F*_*i*_. To estimate the SVD of an entire odor block, we concatenated the *U*_*i*_ for all trials and recalculated the SVD:[*U*_*i*_ … *U*_*m*_] = *USV*^*T*^. As the matrix *U* represented the spatial components of full fluorescence movies, we used the first 40 components of to create a tiff stack could be used to identify glomeruli. Using 8 odors delivered at 2% SVP typically allowed us to identify all glomeruli in the dorsal bulb.

(https://github.com/murselkaradas/ImagingPreProcess)

### Image processing and analysis.

Data analysis was performed using custom-written software in ImageJ (NIH), MATLAB (MathWorks) and the Anaconda Python distribution. Images were aligned to a session-averaged template image using a non-rigid motion correction packagesp. Cellular regions of interest (ROIs) were manually drawn using both mean and maximum intensity projections from 3–5 blocks of trials per session (5–10 min/block). As neurons could be centered in higher or lower z planes, to ensure segmentation of cell somas we also used a z-stack of at least 100 μm in depth centered on the focal plane for ROI selection. During pattern projection, each photostimulation trial exhibited either a trough (due to PMT gating) or a peak (due to high EYFP expression during large pattern projection). To identify the frames in which stimulation occurred for individual ROIs, we calculated the histogram of the fluorescence signal around the onset of stimulation. The value with the most distinct deviation from the mean value was considered to be the stimulation frame. Once the photostimulation frames were identified, they were removed and replaced with an average of the pre- and post-stimulation frames. To reduce noise, we applied a recursive prediction/correction algorithm based on the Kalman filter to trial image stacks (https://imagej.nih.gov/ij/plugins/kalman.html). For Gcamp6f data, we used a gain value of 0.5 and a noise variance of 0.5. For odor and photostimulation trials, we calculated ΔF/F_0_, with F_0_ defined as the average fluorescence 1 s prior to inhalation onset.

### Mitral cell responses to glomerulus stimulation and identifying functional connections.

To identify MTCs functionally connected to individual glomeruli we used a mouse model that combined ChR2 expressed in OSNs (OMP-ChR2-EYFP) and GCamp6f in MTCs (GP.511). First, we identified the anatomical structure of the glomeruli through 2P imaging of the glomerular layer. Then, we stimulated small areas (~70–80% of each glomerulus) targeting individual glomeruli and imaged MTC activity (150–200 μm deeper with respect to the glomerular layer) (Fig. 1D). Photostimulation parameters of 10 ms duration and 15–30 mW/mm^2^ elicited rapid and reliable changes in the fluorescence signal of sister MTCs. Our stimulation duration and spot size were more conservative than previous studies^27,31^ which reduced the number of false positives caused by unwanted excitation through fibers of passage and overlapping glomeruli. We explored the response to off-target photostimulation, targeting a glomerulus as well as several surrounding positions using parameters of 10 ms duration and 20 mW/mm^2^, and determined that unwanted excitation generated smaller responses than direct excitation (Fig. S2A–B). Furthermore, we observed that each glomerulus had a distinct light excitation threshold, and that stimulation of anterior glomeruli could potentially excite some distant posterior glomeruli even at lower light powers (Fig. S2C). Consequently, we adopted the following criteria to identify the parent glomerulus of MTCs: MTCs were assigned to the glomerulus eliciting the largest excitation (mean ΔF/F_0_), or the most posterior glomerulus eliciting a response if there was no significant difference in excitation level (paired t-test). This approach ultimately enabled us to precisely identify sister MTCs.

### Activation onset latency.

To determine the glomerular activation onset latency, we first defined the baseline fluorescence (F_0_) as the mean raw fluorescence signal acquired during the 1-second period preceding odor inhalation. Glomerular responses were then expressed as ΔF/F_0_. A glomerulus was considered responsive, and its latency calculated, only if its mean ΔF/F_0_ response during the first 0.5 seconds post-inhalation was positive. For such responsive glomeruli, onset latency was defined as the first time point at which the ΔF/F_0_ signal exceeded a threshold of 6 standard deviations (SD) above its baseline for four consecutive frames. The SD was computed independently for each glomerulus based on its own pre-stimulus baseline fluorescence fluctuations, and this responsiveness criterion was applied uniformly across all glomeruli. No temporal filtering was applied to the data prior to latency calculation. To ensure distinct response latency values when ranking glomeruli, and to resolve ties among glomeruli with identical calculated latencies, a small adjustment was added to the determined latency: latency (ms) + 1e-3/Fmax (ms), where Fmax represents the maximum response amplitude. This approach for onset latency determination aligns with methods used in previous GCaMP6f imaging studies^33,58,77,78^. For mitral cell responses, the same strategy was employed, but with a lower responsiveness threshold of 4 SD from baseline.

### Odor Similarity.

To determine odor similarity at the glomerular level, we used Spearman’s rank correlation (SciPy package). Non-responsive glomeruli were assigned an identical value equal to the total number of glomeruli imaged, ensuring their contribution to the ranking was uniform. Within-odor similarity (Spearman’s rank correlation) was calculated by correlating responses across different concentrations of the same odor. Across-odor similarity is determined by correlating responses between distinct odor sets, irrespective of concentration. For mitral cell odor-odor correlation, we used the mean mitral cell response during odor presentation (Pearson’s correlation). Final odor-odor correlations were averaged across multiple animals.

### Cell Similarity:

To determine cell similarity, we used cosine similarity. Responses were sampled at approximately 30 Hz (corresponding to 33 ms bins) over a 6-second duration, resulting in a vector of length 180 for each cell. This approach ensures that the time resolution is sufficient to capture fast time-scale differences in responses

### OSN simulation:

Odor inhalation was modeled as a 3 Hz positively rectified sinusoidal signal. The odor concentration developing in the nose (C) over time (t) was described by the differential equation *dC*/*dt* = (*C*_*target*_ − *C*)/*τ*, where τ was set to 100 ms. We simulated (Fig. S4) 1000 olfactory sensory neuron (OSN) response curves using a Hill function for a monomolecular odorant presented at concentration *C*^79^:

$$F_{i,c}=\frac{F_{i,max}}{1+{\left(\frac{1+C/\kappa_{i}}{\eta_{i}C/\kappa_{i}}\right)}^{n_{i}}}$$

Here, *F*_*C*_ represent OSN firing rate, Fmax is the maximum physiologically possible firing rate and *n* the Hill coefficient. The maximal response at saturating concentrations, *F*_∞_ = *F*_*max*_/(1 + *η*^−*n*^) truncated below Fmax by *η*, which controls the equilibrium level of activated receptors. For each OSN receptor, the Fmax is sampled from a gamma distribution *Γ*(α, β), where *α* = 1.5, *β* = 2. The Hill coefficient *n* is sampled uniformly from the range 2 to 4^80^. All other parameters were used as described in^79^. The threshold level for activation is set to 0.1.

### Quantification and statistics.

Data were analyzed using custom-written MATLAB and Python scripts. Statistical analysis of physiological data was performed using standard functions from the Python SciPy library (such as the Kolmogorov-Smirnov test, t test, correlation coefficient, regression). Specific analyses are described alongside experiments in the text and figure captions.

## Supplementary Material

1

## Figures and Tables

**Table 1.** 

Animal model	# mice	Figure #
M72S50 X GP5.11 (Thy1-GCaMP6f)	4	Fig. 5, 6, S11
OMP-ChR2 X GP5.11 (Thy1-GCaMP6f)	4	Fig. 1,2,3E, F, 3, 4, S3,5,7,8,12,13
M72S50 X Ai148 X Tbet-Cre	3	Fig. 1, 3A, B, E, F, S2,9,10
OMP-ChR2 X Ai148 X Tbet-Cre	2	Fig. 3E, F
OMP-ChR2 X Tbet-Cre (AAV5 GCamp8f injection)	1	Fig. 3C, D
Ai93 X OMP-Cre	2	Fig. S6A

**Table 2.** 

Odor name	Abbreviation	Figure #	Air dilution range
2 HydroxyAcetopenone	2HA	4 & 5	0.1 −0.01
Benzaldehyde	BENZ	2,3, 5 & S3,S5,S7	0.05 – 0.0002
Methyl Valerate	MTV	2,3, S3,S5,S7	-
Ethyl Valerate	EVT	2,3, S3,S5,S7	-
2 MethylValerAldehyde	2MVA	2,3, S3,S5,S7	-
2 Methyl 2 Propionate	2MP2P	2,3, S3,S5,S7	-
Hexanal		2,3,S3,S5,S7	-
3- Heptanone	3-HEPN	2,3, S3,S5,S7	-
PhenylPropionate	PhPr	2,3, S3,S5,S7	-
Ethyl Tiglate	ETG	1,2,3,S3,S5,S7	-
Pinene		2,3, S3,S5,S7	-
Heptanoic Acid		2,3, S3,S5,S7	-
IsovalerAldehyde		2,3, S3,S5,S7	-
2-Heptanone	2-HEPN	2,3,S3,S5,S7	-
2 Methyl Butyric Acid	2MBA	2,S5,S7	-
Butyric Acid	BA	2,S5,S7	-
Ethyl Butyrate	EB	2,3,S5,S7	-
Propionic Acid	PA	2,3,S5,S7	-
Trans-CinnamAldehyde	CINALD	2,3, S5,S7	-

## Data Availability

The datasets generated during and/or analyzed during the current study are available from the corresponding author on reasonable request. The preprocessing software is available at https://github.com/murselkaradas/ImagingPreProcess

## References

[1] DiCarlo J. J. & Cox D. D. Untangling invariant object recognition. Trends Cogn Sci 11, 333–341, doi:10.1016/j.tics.2007.06.010 (2007). 17631409

[2] Levick W. R. Receptive fields and trigger features of ganglion cells in the visual streak of the rabbits retina. J. Physiol. 188, 285–307, doi:10.1113/jphysiol.1967.sp008140 (1967). 6032202 PMC1396015

[3] Olveczky B. P., Baccus S. A. & Meister M. Segregation of object and background motion in the retina. Nature 423, 401–408, doi:10.1038/nature01652 (2003). 12754524

[4] Normann R. A. & Perlman I. The effects of background illumination on the photoresponses of red and green cones. J. Physiol. 286, 491–507, doi:10.1113/jphysiol.1979.sp012633 (1979). 439037 PMC1281585

[5] Spors H., Wachowiak M., Cohen L. B. & Friedrich R. W. Temporal dynamics and latency patterns of receptor neuron input to the olfactory bulb. J Neurosci 26, 1247–1259, doi:10.1523/JNEUROSCI.3100-05.2006 (2006). 16436612 PMC6674558

[6] Spors H. & Grinvald A. Spatio-Temporal Dynamics of Odor Representations in the Mammalian Olfactory Bulb. Neuron 34, 301–315 (2002). 11970871 10.1016/s0896-6273(02)00644-x

[7] Wachowiak M. All in a sniff: olfaction as a model for active sensing. Neuron 71, 962–973, doi:10.1016/j.neuron.2011.08.030 (2011). 21943596 PMC3237116

[8] Wesson D. W., Carey R. M., Verhagen J. V. & Wachowiak M. Rapid encoding and perception of novel odors in the rat. PLoS Biol 6, e82, doi:10.1371/journal.pbio.0060082 (2008). 18399719 PMC2288628

[9] Cang J. & Isaacson J. S. In vivo whole-cell recording of odor-evoked synaptic transmission in the rat olfactory bulb. J Neurosci 23, 4108–4116, doi:10.1523/JNEUROSCI.23-10-04108.2003 (2003). 12764098 PMC6741073

[10] Cury K. M. & Uchida N. Robust odor coding via inhalation-coupled transient activity in the mammalian olfactory bulb. Neuron 68, 570–585, doi:10.1016/j.neuron.2010.09.040 (2010). 21040855

[11] Margrie T. W. & Schaefer A. T. Theta oscillation coupled spike latencies yield computational vigour in a mammalian sensory system. J Physiol 546, 363–374, doi:10.1113/jphysiol.2002.031245 (2003). 12527724 PMC2342519

[12] Shusterman R., Smear M. C., Koulakov A. A. & Rinberg D. Precise olfactory responses tile the sniff cycle. Nature Neuroscience 14, 1039–1044, doi:10.1038/nn.2877 (2011). 21765422 PMC13348895

[13] Shepherd G. M. Neuronal Systems Controlling Mitral Cell Excitability. J Physiol 168, 101–117, doi:10.1113/jphysiol.1963.sp007180 (1963). 14056480 PMC1359412

[14] Rall W. & Shepherd G. M. Theoretical reconstruction of field potentials and dendrodendritic synaptic interactions in olfactory bulb. J Neurophysiol 31, 884–915, doi:10.1152/jn.1968.31.6.884 (1968). 5710539

[15] Jahr C. E. & Nicoll R. A. An intracellular analysis of dendrodendritic inhibition in the turtle in vitro olfactory bulb. J Physiol 326, 213–234, doi:10.1113/jphysiol.1982.sp014187 (1982). 7108788 PMC1251469

[16] Yamamoto C., Yamamoto T. & Iwama K. The inhibitory systems in the olfactory bulb studied by intracellular recording. J Neurophysiol 26, 403–415, doi:10.1152/jn.1963.26.3.403 (1963). 14002306

[17] Bathellier B., Buhl D. L., Accolla R. & Carleton A. Dynamic ensemble odor coding in the mammalian olfactory bulb: sensory information at different timescales. Neuron 57, 586–598, doi:10.1016/j.neuron.2008.02.011 (2008). 18304487

[18] Gschwend O. Neuronal pattern separation in the olfactory bulb improves odor discrimination learning. Nature Neuroscience 18, 1474, doi:10.1038/nn.4089 (2015). 26301325 PMC4845880

[19] Yokoi M., Mori K. & Nakanishi S. Refinement of odor molecule tuning by dendrodendritic synaptic inhibition in the olfactory bulb. Proc Natl Acad Sci USA 92, 3371–3375, doi:10.1073/pnas.92.8.3371 (1995). 7724568 PMC42168

[20] Kato H. K., Gillet S. N., Peters A. J., Isaacson J. S. & Komiyama T. Parvalbumin-Expressing Interneurons Linearly Control Olfactory Bulb Output. Neuron 80, 1218–1231, doi:10.1016/j.neuron.2013.08.036 (2013). 24239124 PMC3884945

[21] Olsen S. R., Bhandawat V. & Wilson R. I. Divisive normalization in olfactory population codes. Neuron 66, 287–299, doi:10.1016/j.neuron.2010.04.009 (2010). 20435004 PMC2866644

[22] Roland B. Massive normalization of olfactory bulb output in mice with a ‘monoclonal nose’. Elife 5, doi:10.7554/eLife.16335 (2016). PMC4919110 27177421

[23] Storace D. A. & Cohen L. B. Measuring the olfactory bulb input-output transformation reveals a contribution to the perception of odorant concentration invariance. Nat. Commun. 8, 1–10, doi:10.1038/s41467-017-00036-2 (2017). 28724907 PMC5517565

[24] Niessing J. & Friedrich R. W. Olfactory pattern classification by discrete neuronal network states. Nature 465, 47–52, doi:10.1038/nature08961 (2010). 20393466

[25] Wiechert M. T., Judkewitz B., Riecke H. & Friedrich R. W. Mechanisms of pattern decorrelation by recurrent neuronal circuits. Nat. Neurosci. 13, 1003–1010, doi:10.1038/nn.2591 (2010). 20581841

[26] Chong E. & Rinberg D. Behavioral readout of spatio-temporal codes in olfaction. Curr Opin Neurobiol 52, 18–24, doi:10.1016/j.conb.2018.04.008 (2018). 29694923

[27] Chong E. Manipulating synthetic optogenetic odors reveals the coding logic of olfactory perception. Science 368, doi:10.1126/science.aba2357 (2020). PMC8237706 32554567

[28] Wilson C. D., Serrano G. O., Koulakov A. A. & Rinberg D. A primacy code for odor identity. Nature Communications 8, 1477, doi:10.1038/s41467-017-01432-4 (2017). PMC5684307 29133907

[29] Smear M., Shusterman R., O’Connor R., Bozza T. & Rinberg D. Perception of sniff phase in mouse olfaction. Nature 479, 397–400, doi:10.1038/nature10521 (2011). 21993623

[30] Dana H. Thy1-GCaMP6 transgenic mice for neuronal population imaging in vivo. PLoS One 9, e108697, doi:10.1371/journal.pone.0108697 (2014). 25250714 PMC4177405

[31] Dhawale A. K., Hagiwara A., Bhalla U. S., Murthy V. N. & Albeanu D. F. Nonredundant odor coding by sister mitral cells revealed by light addressable glomeruli in the mouse. Nat Neurosci 13, 1404–1412, doi:10.1038/nn.2673 (2010). 20953197 PMC3208311

[32] Zhu P., Fajardo O., Shum J., Zhang Scharer Y. P. & Friedrich R. W. High-resolution optical control of spatiotemporal neuronal activity patterns in zebrafish using a digital micromirror device. Nat Protoc 7, 1410–1425, doi:10.1038/nprot.2012.072 (2012). 22743832

[33] Short S. M. & Wachowiak M. Temporal Dynamics of Inhalation-Linked Activity across Defined Subpopulations of Mouse Olfactory Bulb Neurons Imaged In Vivo. eNeuro 6, doi:10.1523/ENEURO.0189-19.2019 (2019). PMC6597857 31209151

[34] Haddad R. Olfactory cortical neurons read out a relative time code in the olfactory bulb. Nature Neuroscience 16, 949, doi:10.1038/nn.3407 (2013). 23685720 PMC3695490

[35] Hopfield J. J. Pattern recognition computation using action potential timing for stimulus representation. Nature 376, 33–36, doi:10.1038/376033a0 (1995). 7596429

[36] Schaefer A. T. & Margrie T. W. Spatiotemporal representations in the olfactory system. Trends Neurosci 30, 92–100, doi:10.1016/j.tins.2007.01.001 (2007). 17224191

[37] Zhang J., Huang G., Dewan A., Feinstein P. & Bozza T. Uncoupling stimulus specificity and glomerular position in the mouse olfactory system. Molecular and Cellular Neuroscience 51, 79–88, doi:10.1016/j.mcn.2012.08.006 (2012). 22926192 PMC3494770

[38] Arneodo E. M. Stimulus dependent diversity and stereotypy in the output of an olfactory functional unit. Nature Communications 9, 1347, doi:10.1038/s41467-018-03837-1 (2018). PMC5890244 29632302

[39] Liu S., Puche A. C. & Shipley M. T. The Interglomerular Circuit Potently Inhibits Olfactory Bulb Output Neurons by Both Direct and Indirect Pathways. J. Neurosci. 36, 9604–9617, doi:10.1523/JNEUROSCI.1763-16.2016 (2016). 27629712 PMC5039244

[40] Whitesell J. D., Sorensen K. A., Jarvie B. C., Hentges S. T. & Schoppa N. E. Interglomerular Lateral Inhibition Targeted on External Tufted Cells in the Olfactory Bulb. J. Neurosci. 33, 1552–1563, doi:10.1523/JNEUROSCI.3410-12.2013 (2013). 23345229 PMC3711647

[41] Mombaerts P. Targeting olfaction. Curr Opin Neurobiol 6, 481–486, doi:10.1016/s0959-4388(96)80053-5 (1996). 8794106

[42] Aungst J. L. Centre–surround inhibition among olfactory bulb glomeruli. Nature 426, 623–629, doi:10.1038/nature02185 (2003). 14668854

[43] Banerjee A. An Interglomerular Circuit Gates Glomerular Output and Implements Gain Control in the Mouse Olfactory Bulb. Neuron 87, 193–207, doi:10.1016/j.neuron.2015.06.019 (2015). 26139373 PMC4633092

[44] De Saint Jan D., Hirnet D., Westbrook G. L. & Charpak S. External tufted cells drive the output of olfactory bulb glomeruli. J Neurosci 29, 2043–2052, doi:10.1523/JNEUROSCI.5317-08.2009 (2009). 19228958 PMC6666334

[45] Gire D. H. Mitral cells in the olfactory bulb are mainly excited through a multistep signaling path. J Neurosci 32, 2964–2975, doi:10.1523/JNEUROSCI.5580-11.2012 (2012). 22378870 PMC3467005

[46] Miyamichi K. Dissecting local circuits: parvalbumin interneurons underlie broad feedback control of olfactory bulb output. Neuron 80, 1232–1245, doi:10.1016/j.neuron.2013.08.027 (2013). 24239125 PMC3932159

[47] Arenkiel B. R. In vivo light-induced activation of neural circuitry in transgenic mice expressing channelrhodopsin-2. Neuron 54, 205–218, doi:10.1016/j.neuron.2007.03.005 (2007). 17442243 PMC3634585

[48] Lehmann A., D’Errico A., Vogel M. & Spors H. Spatio-Temporal Characteristics of Inhibition Mapped by Optical Stimulation in Mouse Olfactory Bulb. Front Neural Circuits 10, 15, doi:10.3389/fncir.2016.00015 (2016). 27047340 PMC4801895

[49] Bolding K. A. & Franks K. M. Recurrent cortical circuits implement concentration-invariant odor coding. Science 361, eaat6904, doi:10.1126/science.aat6904 (2018). PMC6492549 30213885

[50] Koldaeva A., Schaefer A. T. & Fukunaga I. Rapid task-dependent tuning of the mouse olfactory bulb. Elife 8, doi:10.7554/eLife.43558 (2019). PMC6365054 30724732

[51] Trejo D. H. Fast updating feedback from piriform cortex to the olfactory bulb relays multimodal reward contingency signals during rule-reversal. bioRxiv, doi:10.1101/2023.09.12.557267 (2023). PMC11754465 39843439

[52] Yamada Y. Context- and Output Layer-Dependent Long-Term Ensemble Plasticity in a Sensory Circuit. Neuron 93, 1198–1212 e1195, doi:10.1016/j.neuron.2017.02.006 10.1016/j.neuron.2017.02.006. Epub 2017 Feb 23. (2017). 28238548 PMC5352733

[53] Li W. L. Adult-born neurons facilitate olfactory bulb pattern separation during task engagement. Elife 7, doi:10.7554/eLife.33006 (2018). PMC5912906 29533179

[54] Wilson C. D. Rapid encoding of odor identity via a primacy code pHD thesis, NYU Langone Health, (2018).

[55] Giaffar H., Shuvaev S., Rinberg D. & Koulakov A. A. The primacy model and the structure of olfactory space. bioRxiv, doi:10.1101/255661 (2023). PMC11423968 39255274

[56] Uchida N. & Mainen Z. F. Speed and accuracy of olfactory discrimination in the rat. Nat Neurosci 6, 1224–1229, doi:10.1038/nn1142 (2003). 14566341

[57] Uchida N. & Mainen Z. F. Odor concentration invariance by chemical ratio coding. Front Syst Neurosci 1, 3, doi:10.3389/neuro.06.003.2007 (2007). 18958244 PMC2526272

[58] Economo M. N., Hansen K. R. & Wachowiak M. Control of Mitral/Tufted Cell Output by Selective Inhibition among Olfactory Bulb Glomeruli. Neuron 91, 397–411, doi:10.1016/j.neuron.2016.06.001 (2016). 27346531 PMC6474342

[59] Friedrich R. W. Neuronal computations in the olfactory system of zebrafish. Annu. Rev. Neurosci. 36, 383–402, doi:10.1146/annurev-neuro-062111-150504 10.1146/annurev-neuro-062111-150504. Epub 2013 May 29. (2013). 23725002

[60] Friedrich R. W. & Laurent G. Dynamics of olfactory bulb input and output activity during odor stimulation in zebrafish. J. Neurophysiol. 91, 2658–2669, doi:10.1152/jn.01143.2003 (2004). 14960561

[61] Chou Y. H., Zheng X., Beachy P. A. & Luo L. Patterning axon targeting of olfactory receptor neurons by coupled hedgehog signaling at two distinct steps. Cell 142, 954–966, doi:10.1016/j.cell.2010.08.015 (2010). 20850015 PMC3028148

[62] Hong E. J. & Wilson R. I. Simultaneous encoding of odors by channels with diverse sensitivity to inhibition. Neuron 85, 573–589, doi:10.1016/j.neuron.2014.12.040 (2015). 25619655 PMC5495107

[63] Wilson R. I., Turner G. C. & Laurent G. Transformation of olfactory representations in the Drosophila antennal lobe. Science 303, 366–370, doi:10.1126/science.1090782 (2004). 14684826

[64] Chae H., Banerjee A., Dussauze M. & Albeanu D. F. Long-range functional loops in the mouse olfactory system and their roles in computing odor identity. Neuron 110, 3970–3985 e3977, doi:10.1016/j.neuron.2022.09.005 (2022). 36174573 PMC9742324

[65] Fukunaga I., Berning M., Kollo M., Schmaltz A. & Schaefer A. T. Two Distinct Channels of Olfactory Bulb Output. Neuron 75, 320–329, doi:10.1016/j.neuron.2012.05.017 (2012). 22841316

[66] Chen Y. High-throughput sequencing of single neuron projections reveals spatial organization in the olfactory cortex. Cell 185, 4117–4134 e4128, doi:10.1016/j.cell.2022.09.038 (2022). 36306734 PMC9681627

[67] Kikuta S., Fletcher, Max L., Homma R., Yamasoba T. & Nagayama S. Odorant Response Properties of Individual Neurons in an Olfactory Glomerular Module. Neuron 77, 1122–1135, doi:10.1016/j.neuron.2013.01.022 (2013). 23522047 PMC3607817

[68] Tan J., Savigner A., Ma M. & Luo M. Odor information processing by the olfactory bulb analyzed in gene-targeted mice. Neuron 65, 912–926, doi:10.1016/j.neuron.2010.02.011 (2010). 20346765 PMC2901914

[69] Rinberg D., Koulakov A. & Gelperin A. Sparse odor coding in awake behaving mice. J Neurosci 26, 8857–8865, doi:10.1523/JNEUROSCI.0884-06.2006 (2006). 16928875 PMC6674368

[70] Kato H. K., Chu M. W., Isaacson J. S. & Komiyama T. Dynamic Sensory Representations in the Olfactory Bulb: Modulation by Wakefulness and Experience. Neuron 76, 962–975, doi:10.1016/j.neuron.2012.09.037 (2012). 23217744 PMC3523713

[71] Osborne J. E. & Dudman J. T. RIVETS: a mechanical system for in vivo and in vitro electrophysiology and imaging. PLoS One 9, e89007, doi:10.1371/journal.pone.0089007 (2014). 24551206 PMC3925229

[72] Pologruto T. A., Sabatini B. L. & Svoboda K. ScanImage: flexible software for operating laser scanning microscopes. Biomed. Eng. Online 2, 13, doi:10.1186/1475-925X-2-13 (2003). 12801419 PMC161784

[73] Jennings L., Williams E., Caton S., Avlas M. & Dewan A. Estimating the relationship between liquid- and vapor-phase odorant concentrations using a photoionization detector (PID)-based approach. Chem. Senses 48, doi:10.1093/chemse/bjac038 (2023). PMC10208748 36571813

[74] Gill J. V. Precise Holographic Manipulation of Olfactory Circuits Reveals Coding Features Determining Perceptual Detection. Neuron 108, 382–393 e385, doi:10.1016/j.neuron.2020.07.034 (2020). 32841590 PMC8289117

[75] Pnevmatikakis E. A. & Giovannucci A. NoRMCorre: An online algorithm for piecewise rigid motion correction of calcium imaging data. J. Neurosci. Methods 291, 83–94, doi:10.1016/j.jneumeth.2017.07.031 (2017). 28782629

[76] Stringer C. Spontaneous behaviors drive multidimensional, brainwide activity. Science 364, 255, doi:10.1126/science.aav7893 (2019). 31000656 PMC6525101

[77] Homma R. Narrowly Confined and Glomerulus-Specific Onset Latencies of Odor-Evoked Calcium Transients in the Juxtaglomerular Cells of the Mouse Main Olfactory Bulb. eNeuro 6, doi:10.1523/ENEURO.0387-18.2019 (2019). PMC6397951 30834302

[78] Sato T., Homma R. & Nagayama S. Direct comparison of odor responses of homologous glomeruli in the medial and lateral maps of the mouse olfactory bulb. eNeuro 7, ENEURO.0449-0419.2020, doi:10.1523/ENEURO.0449-19.2020 (2020). PMC7073388 31974110

[79] Reddy G., Zak J. D., Vergassola M. & Murthy V. N. Antagonism in olfactory receptor neurons and its implications for the perception of odor mixtures. Elife 7, doi:10.7554/eLife.34958 (2018). PMC5915184 29687778

[80] Zak J. D., Reddy G., Vergassola M. & Murthy V. N. Antagonistic odor interactions in olfactory sensory neurons are widespread in freely breathing mice. Nat. Commun. 11, 1–12, doi:10.1038/s41467-020-17124-5 (2020). 32620767 PMC7335155

