# Supplementary material for: Rapid temporal processing in the olfactory bulb underlies concentration invariant odor identification and signal decorrelation
Source: Res Sq. 2025 Jul 4:rs.3.rs-4415331. Preprint. [Version 1] doi: 10.21203/rs.3.rs-4415331/v1 (PMC12236928; doi:10.21203/rs.3.rs-4415331/v1)
Supplement: 1 [file NIHPPRS4415331V1-supplement-1.pdf]

Supplemental Figures:

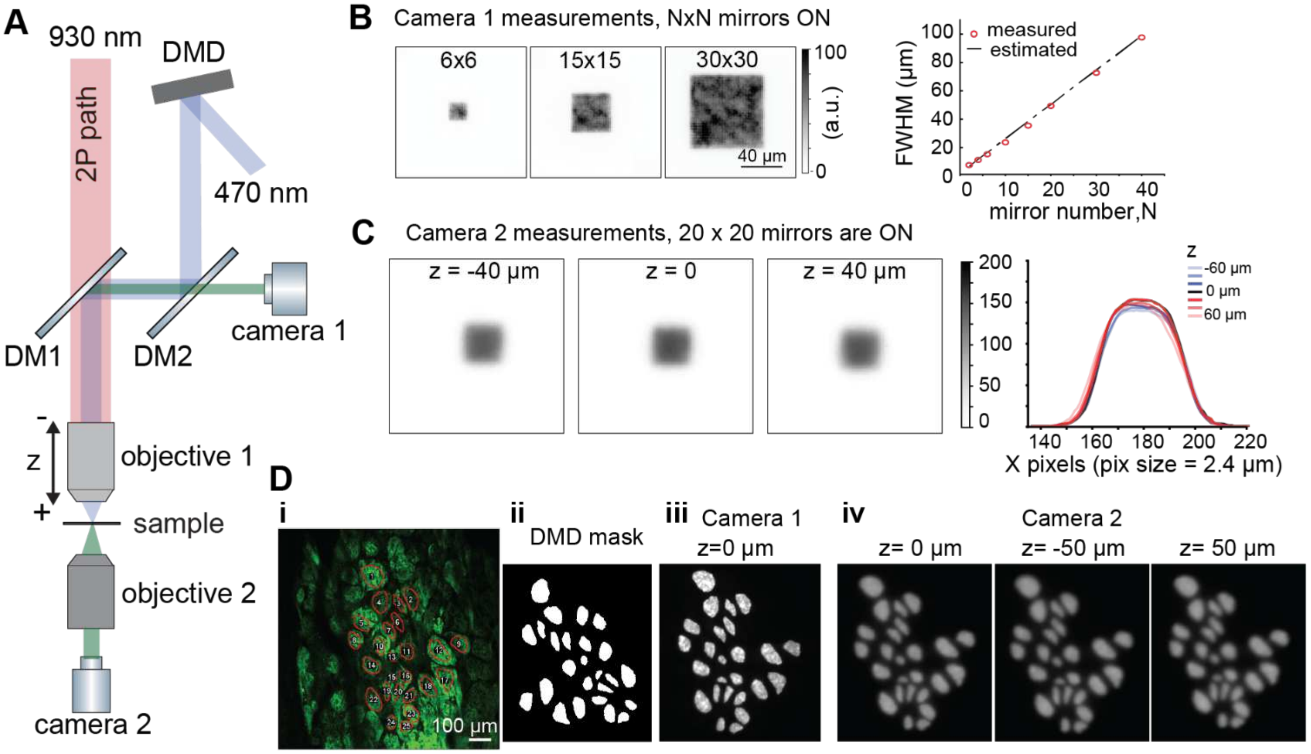

Figure S1. Microscope design and characterization. **A.** 2P microscope equipped with a DMD-based pattern stimulation setup and wide field cameras for alignment calibration. Camera 1 was positioned in the conjugate plane of the DMD and was used for alignment of the DMD projected patterns to the 2P imaging. Camera 2 and Objective 2 are temporarily placed below the sample plane and used to characterize the axial spread of projected patterns. DM - dichoric mirror. **B.** *Left:* Images of illuminated areas of different size square patches (NxN) of active DMD pixels taken by Camera 1. *Right:* Estimated and measured full-width half-maximum (FWHM) of illuminated areas for different patch sizes. **C.** *Left:* Images of projected patterns of 20x20 DMD pixels at different positions of the Objective 1 taken by Camera 2. *Right:* Intensity profiles of projected patterns along the x-axis. **Di**. 2P images of the glomerular layer with manually drawn glomerular ROIs. **Dii**. Corresponding DMD mask patterns; **Diii.** Image of the glomerular mask pattern with Camera 1. **Div.** Images of the glomerular mask pattern with Camera 2 at different position of the Objective 1.

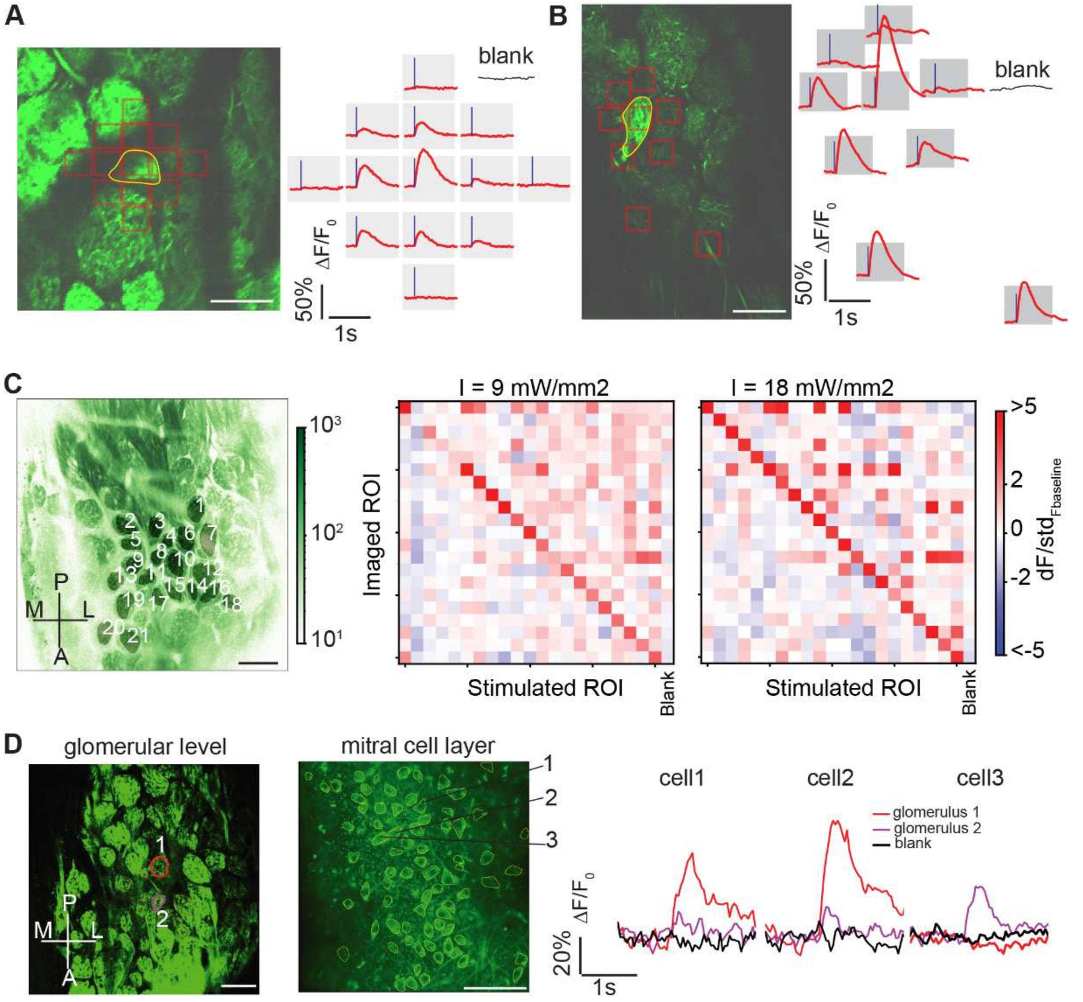

Figure S2. Specificity of stimulation. **A.** *Left:* Glomerular layer image with a pattern of stimulated spots around a target glomerulus. The ROI of the target glomerulus is shown in yellow. *Right:* The target glomerulus responses to individual spot stimulation (10 ms, 18 mW/mm^2), measured using 2P Ca^{2+}-imaging in the plane of stimulation (mean of 5 photostimulation repetitions). **B.** The same as **A** for stimulation spots placed at different locations to evaluate the effect of stimulating axons-of-passage (mean of 5 photostimulation repetitions). **C.** *Left:* ROIs of multiple glomeruli, labeled 1-21 along the posterior-anterior axis. *Right:* Heatmaps of multiple glomerular responses to stimulation of different ROIs for two stimulation intensities: 9 and 18 mW/mm^2 (mean of 10 photostimulation repetition, n = 21 glomeruli). Note: for higher stimulation intensities, one could observe more active glomeruli in the upper right corner of the heatmap, presumably due to activation of axons of passage. Stimulation of ROIs with a higher number (i.e, more anterior) led to off-target activation of glomeruli with lower numbers (i.e., more posterior). **D.** *Left:* ROIs of two glomeruli. *Middle:* image of mitral cells 200 µm below the targeted area. *Right:* Responses of three mitral cells to stimulation of ROIs corresponding to two glomeruli. Note: Stimulation of glomerulus 1 (more posterior) evoked strong responses for Cell 1 and 2 (presumably D-MTCs) but stimulating glomerulus 2 (more anterior) evoked responses for Cell 3 (presumably D-MTC) as well as off-target responses for Cells 1 and 2 (mean of 10 photostimulation repetitions).

(A&B collected from M72S50 x Thy1-GCaMP6f and C&D OMP-ChR2 x Thy1-GCaMP6f).

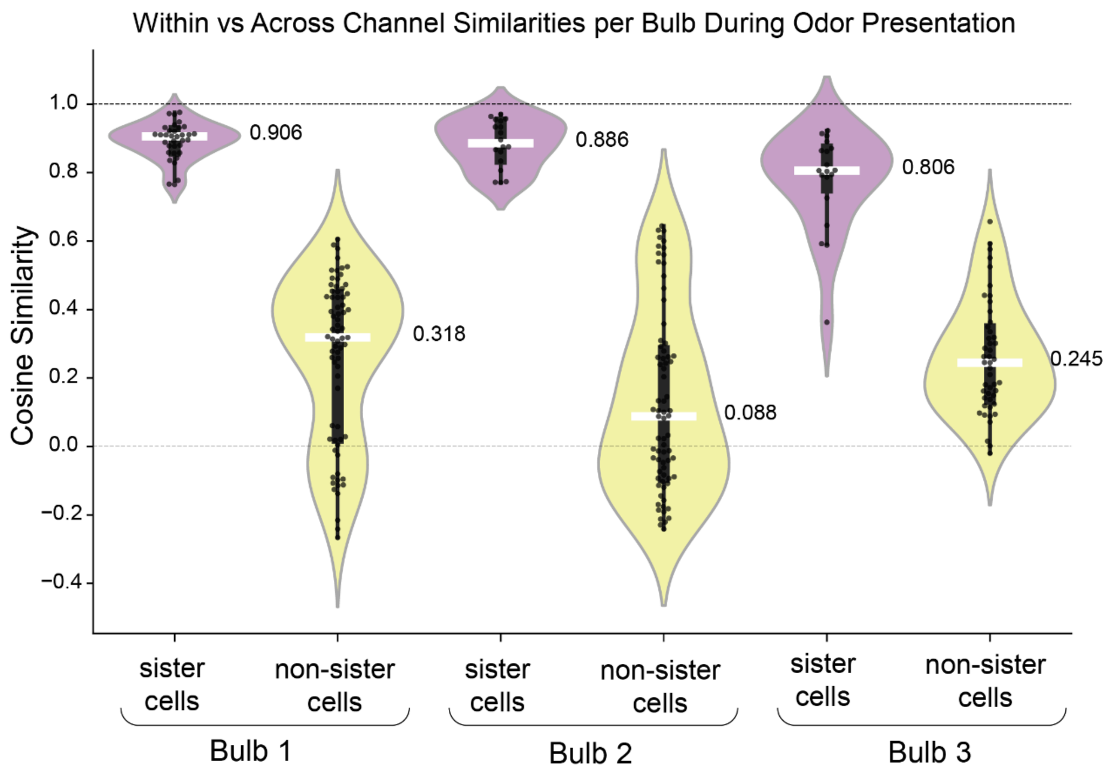

Figure S3: Stereotypy of responses in intra-glomerular channels for each bulb. Violin plots summarizing the distribution of cosine similarity values for intra-glomerular (purple) and inter-glomerular (yellow) MTC pairs during the odor phases. (n = 71 intra-glomerular and 749 inter-glomerular pairs in 2 animals). Mean cosine similarities were as follows intra-glomerular: 0.906, 0.886, 0.806; inter-glomerular: 0.318, 0.088, 0.245.

Modeling of concentration related changes of onset latency ranking

Odor inhalation was modeled as a 3 Hz positively rectified sinusoidal signal. The odor concentration developing in the nose (C) over time (t) was described by the differential equation $dC/dt = (C_{target} - C)/\tau$, where τ was set to 100 ms. We simulated (**Fig. S4**) 1000 olfactory sensory neuron (OSN) response curves using a Hill function for a monomolecular odorant presented at concentration C [1]:

$$F_{i,c} = \frac{F_{i,max}}{1 + \left(\frac{1 + C/\kappa_i}{\eta_i C/\kappa_i}\right)^{n_i}}$$

Here, $F_{i,c}$ represent OSN firing rate, $F_{i,max}$ is the maximum physiologically possible firing rate and n_i the Hill coefficient, κ_i is control the affinity of odor to the receptor. The maximal response at saturating concentrations, $F_\infty = F_{max}/(1 + \eta^{-n})$ truncated below F_{max} by η, which controls the equilibrium level of activated receptors. For each OSN receptor, the F_{max} is sampled from a gamma distribution $\Gamma(\alpha, \beta)$, where $\alpha = 1.5, \beta = 2$. The Hill coefficient n is sampled uniformly from the range 2 to 4 [2]. All other parameters were used as described in [1]. The threshold level for activation is set to 0.1.

The change of an onset latency rank may occur due to an interplay between slopes of receptor-ligand dose response curve and a rate of the concentration increase in the nose. Receptors with a relatively shallow dose response slopes are much stronger affected by the rate of concentration increase than those with a steep slope, which may lead to a swap of activation onset ranking between such receptors.

1 Reddy, G., Zak, J. D., Vergassola, M. & Murthy, V. N. Antagonism in olfactory receptor neurons and its implications for the perception of odor mixtures. *Elife* **7**, doi:10.7554/eLife.34958 (2018).

2 Zak, J. D., Reddy, G., Vergassola, M. & Murthy, V. N. Antagonistic odor interactions in olfactory sensory neurons are widespread in freely breathing mice. *Nat. Commun.* **11**, 1-12, doi:10.1038/s41467-020-17124-5 (2020).

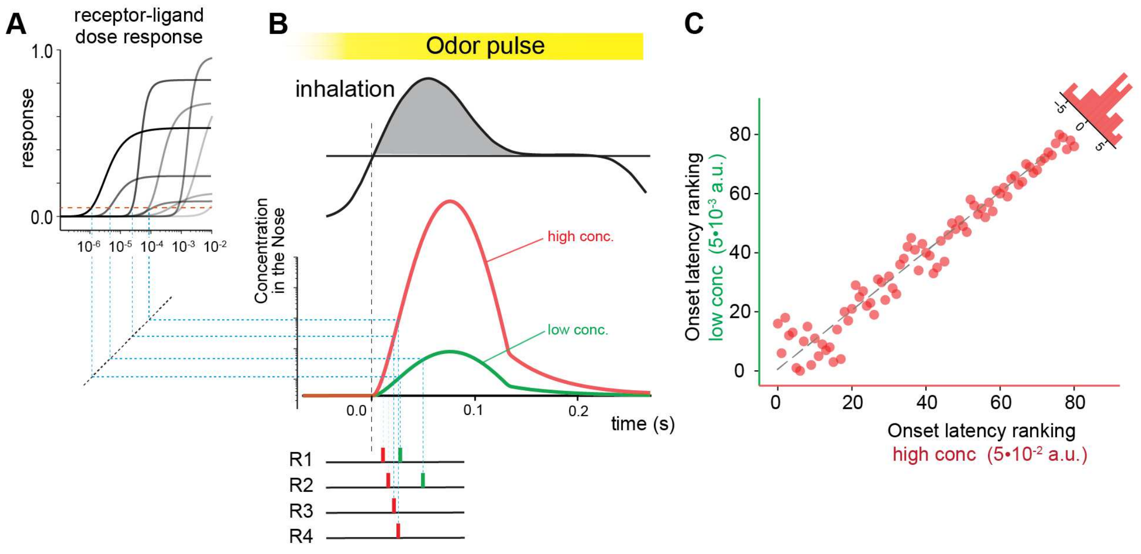

Figure S4. **Modeling of odor concentration dynamics and activation onset ranking**. **B.** Examples of receptor-ligand dose response curves (following [1]). **A.** Schematics of a respiration cycle (*top panel*) and the temporal profiles of odor concentration in the nose for low (green) and high (red) concentrations (*middle panel*). Schematics of the temporal sequences of receptor activation for low and high odor concentrations (*bottom panel*). Individual receptors become activated when the concentration in the nose crosses a specific threshold (red dashed line at panel **A**). **B.** Receptors-ligand dose response curve for a specific odor (see text below for details). **C.** Scatter plot of activation onset ranking with fixed threshold level at concentration $5 \cdot 10^{-2}$ a.u. versus rank at concentration $5 \cdot 10^{-3}$ a.u., demonstrating rank swapping across concentrations using a simple thresholding (threshold level =0.1) method.

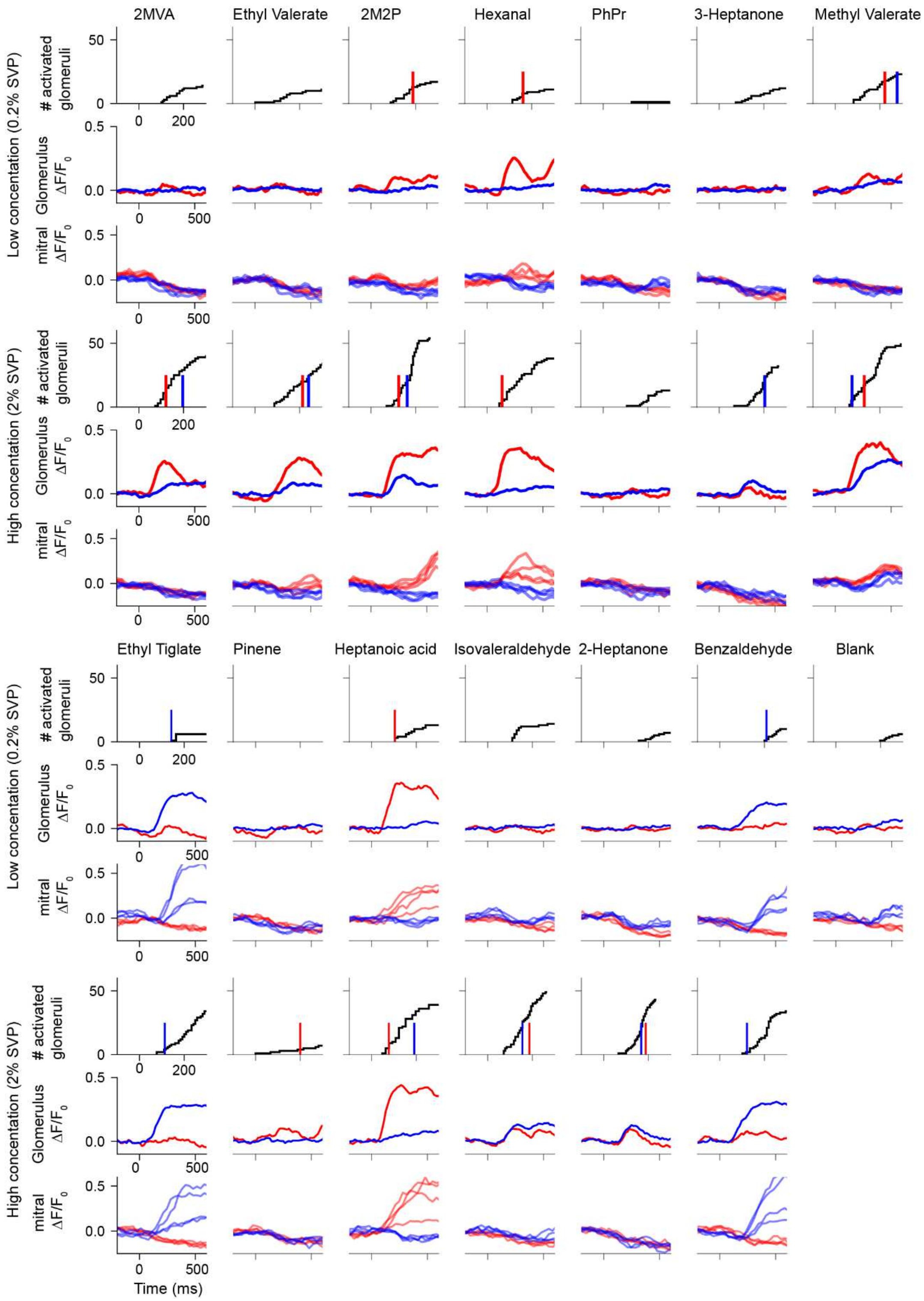

Figure S5. **Concentration dependence of glomerular and D-MTC responses.** Cumulative distribution of glomerulus activation response latencies relative to the onset of inhalation for 13 odors and blank control (mean latency for 10 odor presentations, n = 90 glomeruli). The latencies of two glomeruli from each panel are shown by vertical bars (red and blue). The Ca^{2+} responses of these glomeruli (blue and red traces) and their D-MTCs (blue and red traces, mean of 10 odor presentations, n = 4 cells/glomerulus in 1 mouse). (All presented data collected from mouse line: OMP-ChR2 x Thy1-GCaMP6f).

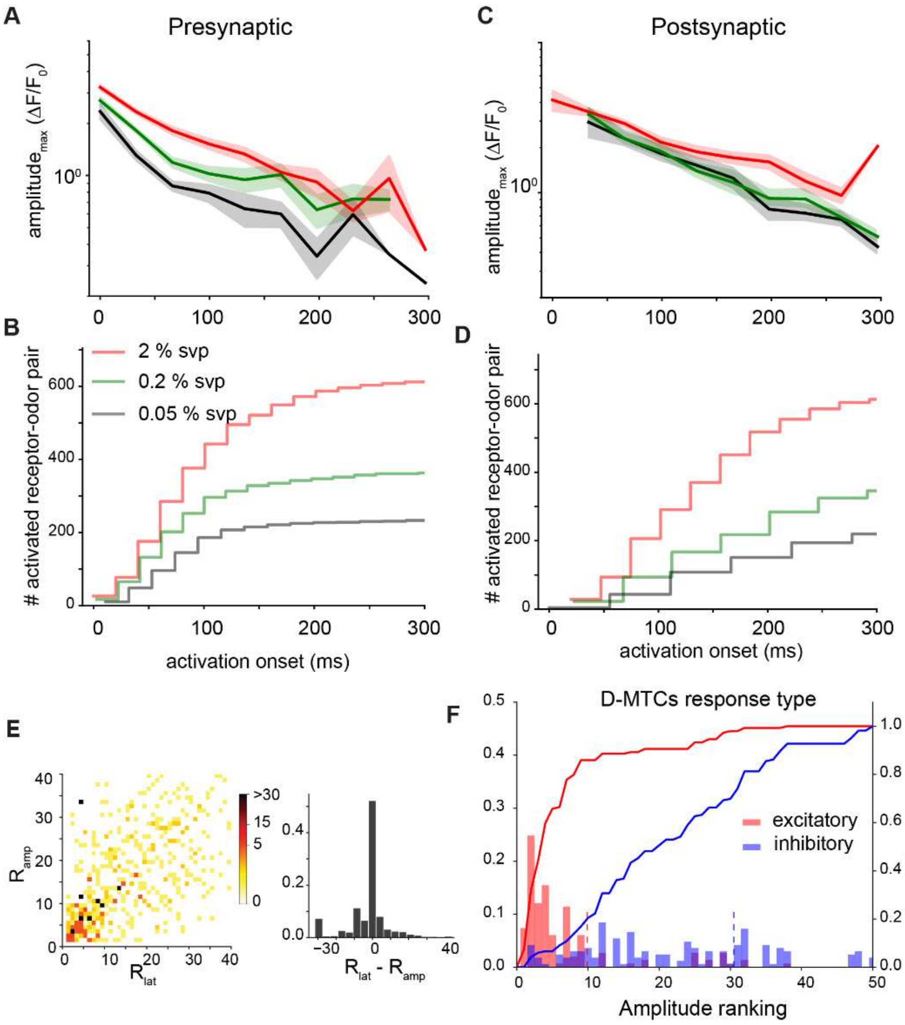

Figure S6. A. A scatter plot of glomerular activation onset vs response amplitude for three different concentrations (0.05% SVP - black, 0.2% - green, and 2% - red) of 10 odors measured at the OSN (presynaptic, OMP-Cre x GcaMP6f) level (low conc. slope=-5.9e-3 log(ΔF/F0)/ms, R^2 = 0.86; mid conc.: slope = -4.7e-3, R^2 = 0.82; high conc.: slope= 5.9e-3, R^2=0.90 mean of 5 repetitions for 121 glomeruli in two bulbs). Lines are average dependencies (30 ms binning) and shades are standard error mean **B.** The number of activated receptor-odor pairs as a function of time after inhalation onset for three odor concentrations. **C.** and **D.** The same as **A** and **B** for postsynaptic glomerular responses (low conc. slope=-6.9e-3, R^2=0.98; mid conc.: slope=-6.8e-3; R^2 = 0.97; high conc.: slope=-3.8e-3, R^2=0.71, mean of 5 repetitions for 228 glomeruli in 3 mice). **E**. *Left panel:* Glomerular activation amplitude ranking versus latency ranking for 1120 odor-glomerulus pairs, measured postsynaptically. *Right panel:* Distribution of differences between amplitude and latency ranking. **F.** Response type distribution for D-MTCs of 9 glomeruli (32 D-MTCs) to 13 odors/2 concentrations for glomerulus response amplitude-based ranking (mean of 5 repetitions in 1 mouse 2 bulbs).

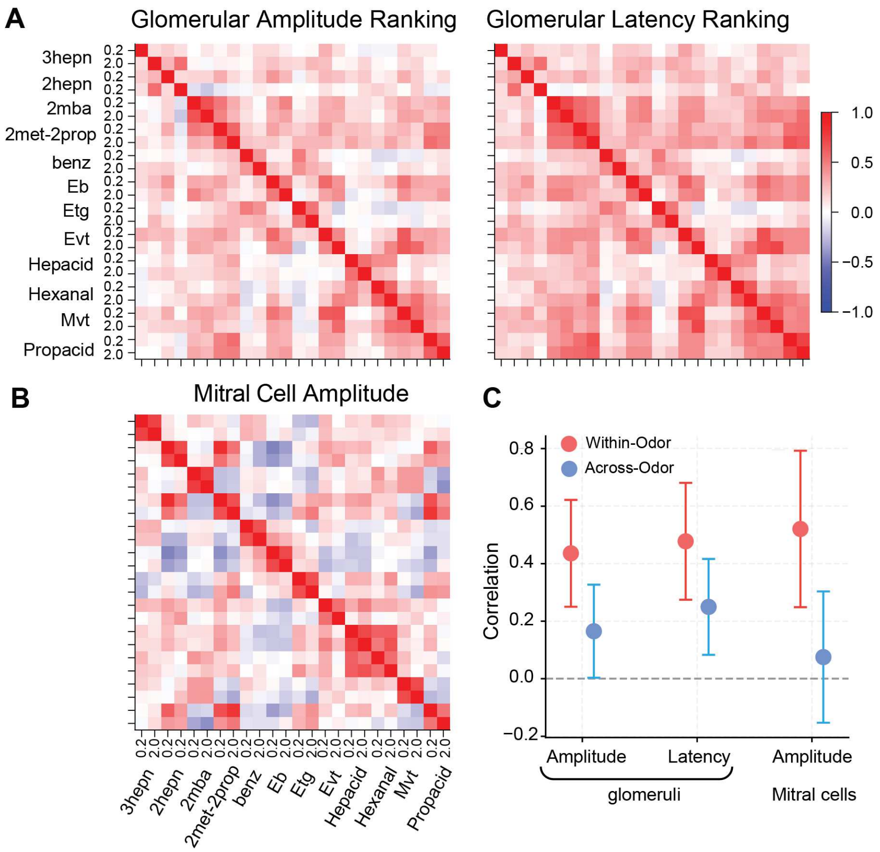

Figure S7. Odor-odor correlation at different level processing. **A**. Glomerular odor-odor correlations, calculated using amplitude ranking (left) and onset latency ranking (right) for 12 distinct odors at 2 concentrations for each odor. **B**. Mitral cell response similarity for the same 12 odors. **C**. Summary statistics depicting mean ± standard deviation of correlation values for within-odor (across concentrations) and across-odor (different odors) comparisons: Data were acquired from 12 odors presented at two concentrations (0.2% and 2% SVP) across three mice. Glomerular signals were recorded with 3-5 trials per odor/concentration, while mitral cell responses were collected with 8-10 trials per odor/concentration. (All presented data collected from mouse line: OMP-ChR2 x Thy1-GCaMP6f).

Important observations: **1)** correlations between different odors are significantly lower than for the same odors at different concentrations (**C**) (Glomerular level correlation: within odor: 0.44±0.19, across: odor: 0.17±0.17, t=5.42, p<1e-4). This difference is increased through the transition from the glomerular to MTC level (Mitral level odor correlation: within odor: 0.49±0.23, across: odor: 0.09±0.22; t=6.43, p<1e-4). **2)** The correlation between the same odors at different concentrations slightly increases at MTC level, emphasizing concertation invariant odor representation, while correlations across different odors decreases, which supports the decorrelation hypothesis (two sample t-test, t=4.61, p<1e-4).

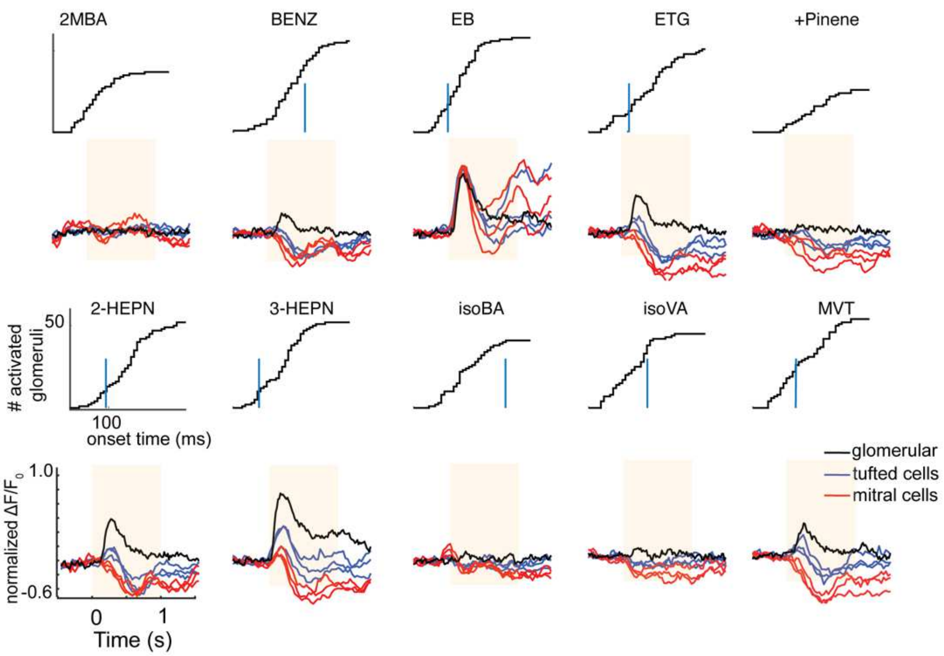

Figure S8. Cumulative distribution of glomerulus activation response latencies relative to the onset of inhalation for 8 odors (Top row and 3rd row, mean latency for 10 odor presentations, ~2%SVP, n = 67 glomeruli in a mice). The onset time of a glomerulus is shown by vertical blue bars. Mean response traces for the same glomerulus (black) and its connected mitral (red) and tufted (blue) cells (second and fourth rows, mean of 10 odor presentations, ~2 %SVP, n = 3 mitral and 3 tufted cells in a mouse.
(All presented data collected from mouse line: OMP-ChR2 x Thy1-GCaMP6f).

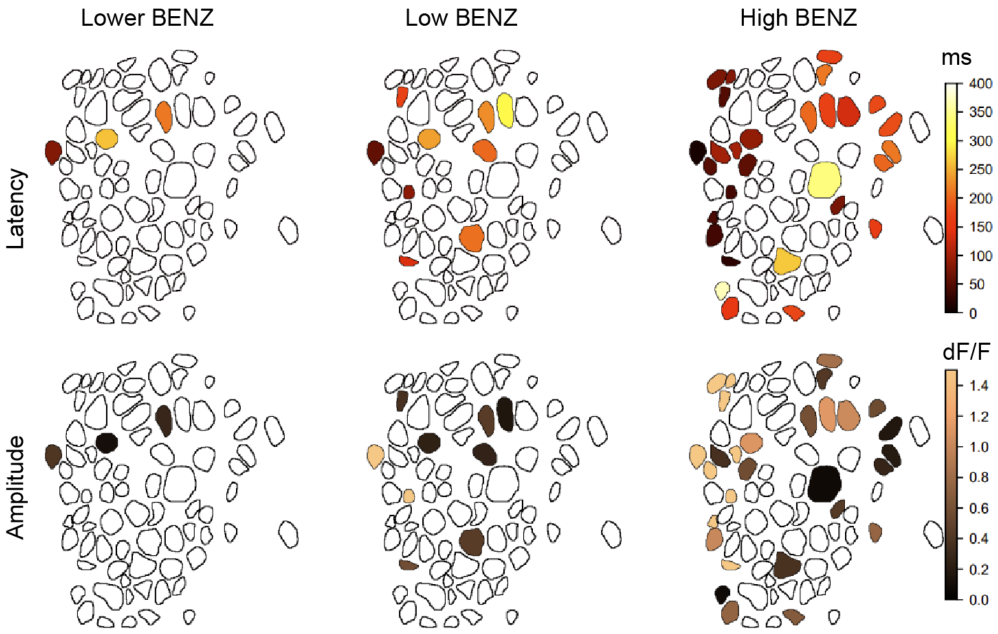

Figure S9. Spatial distribution of olfactory bulb responses to benzaldehyde (BENZ) concentrations. (Top row) Latency maps showing response latencies (ms) for lower BENZ, low BENZ, and high BENZ conditions. (Bottom row) Amplitude maps showing response amplitudes (dF/F) for the same conditions. (lower: 0.02%SVP, low: 0.2%SVP, high: 2%SVP).
(All presented data collected from mouse line: M72S50 x TbetCre-GCaMP6f).

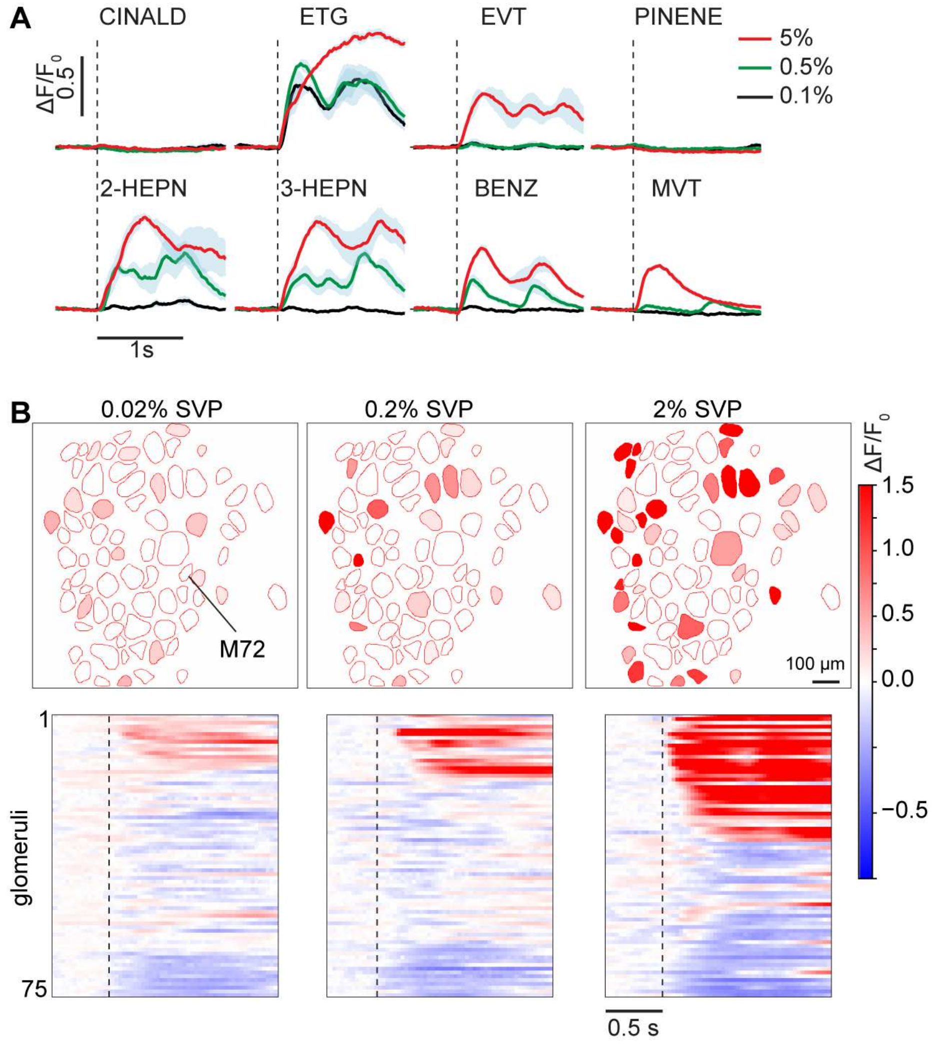

Figure S10. A. Temporal profiles of Ca^{2+} responses of the M72 glomerulus for 8 odors at 3 different concentrations (reported as %SVP in legend, mean of 10 odor presentations, n = 5 glomeruli in a mouse). **B.** Spatial map of glomerular responses (top) and the correspondent temporal profiles (bottom) to 3 concentrations of BENZ (mean of 10 odor presentations, n = 75 glomeruli in a mouse).
(All presented data collected from mouse line: M72S50 x TbetCre-GCaMP6f).

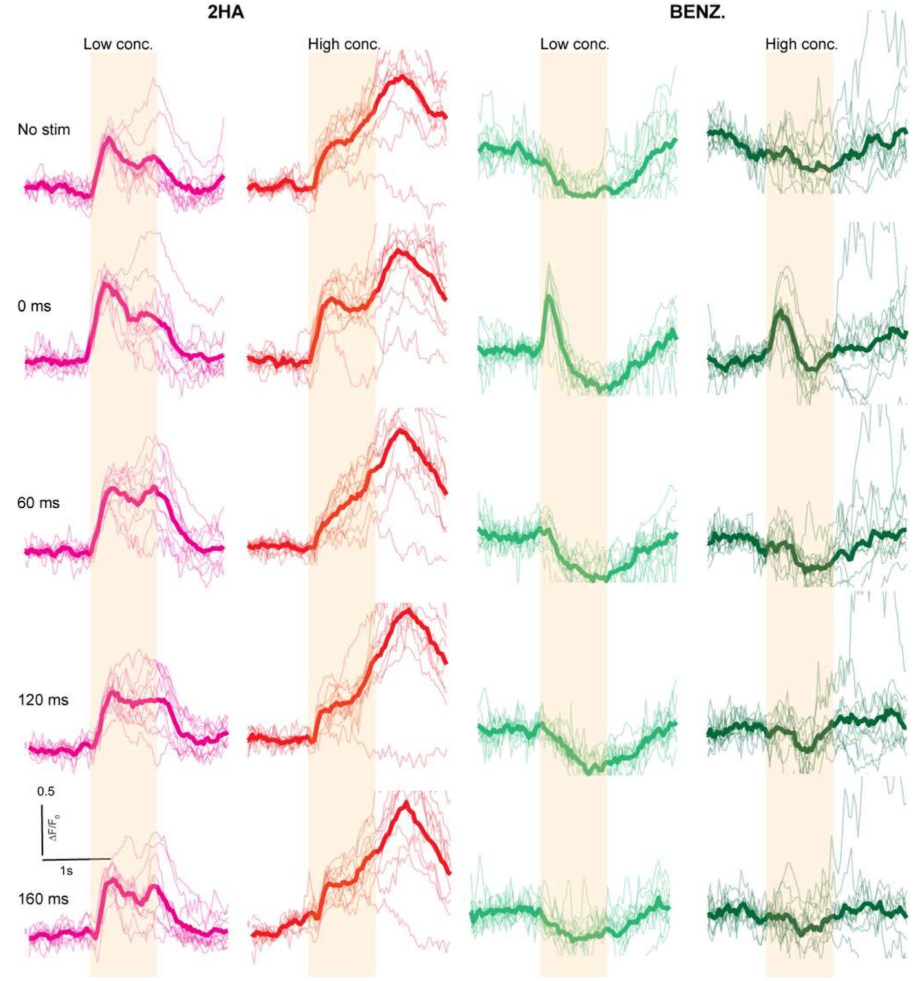

Figure S11. Individual M72 D-MTC responses. Mean amplitude of D-MTC responses to a light pulse as a function of pulse timing relative to the onset of inhalation. Pulses were delivered with the presentation of 2HA, a strong ligand of M72 receptor (red), and with BNZ, a weak ligand of M72 receptor, at low (light green) and high (dark green) concentrations (mean of 5 photostimulations, 10 ms, ~20 mW/mm^2, n = 11 D-MTCs across 4 mice).
(All presented data collected from mouse line: M72S50 x Thy1-GCaMP6f).

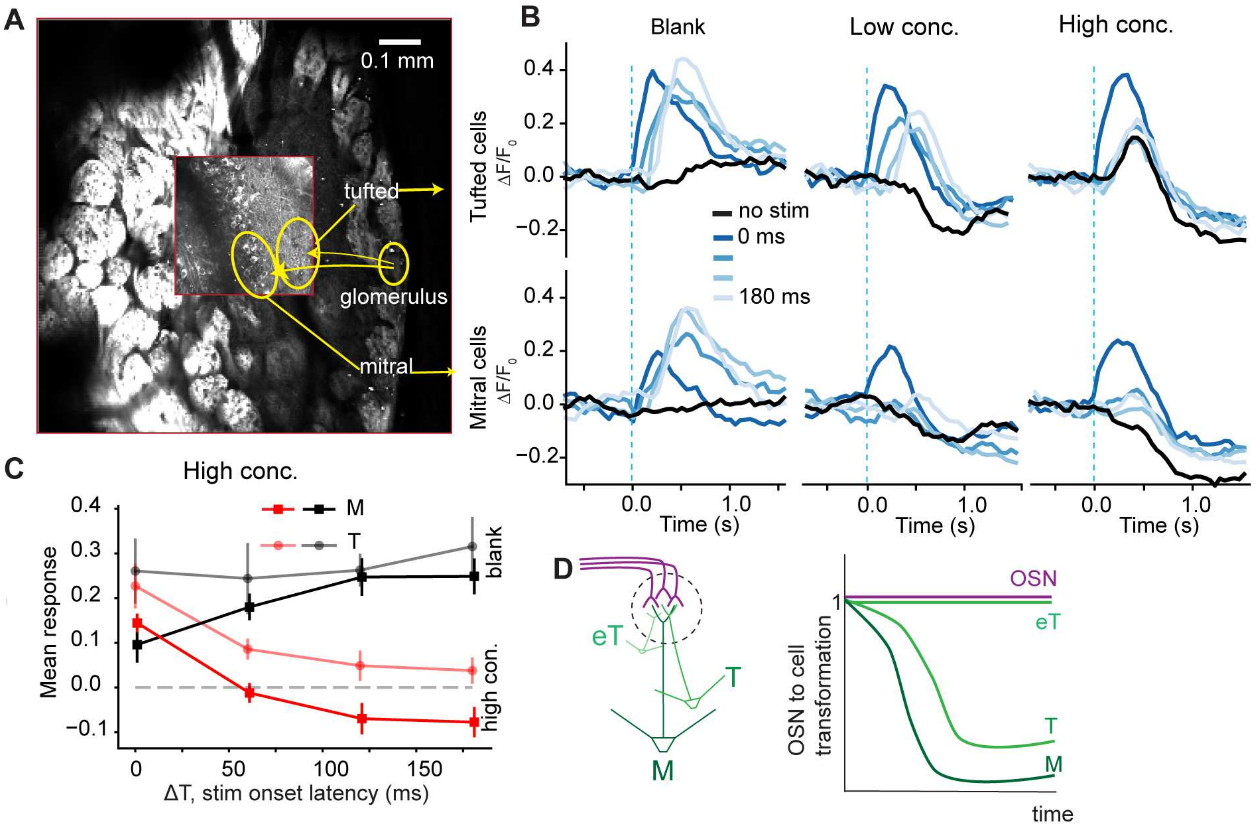

Figure S12: Weak Ligand Results for a Generic Glomerulus, Tufted vs Mitral Cells. A. Large-field view of the glomerular layer with a targeted glomerulus near the midline and an image of the MTC layer, with identified D-MTCs connected to the target glomerulus. Putative tufted and mitral cells were identified in the same imaging plane at approximately 150 µm deeper than the glomerular layer (see inset in the middle). Cells were classified as mitral and tufted based on local anatomy, with tufted cell somas situated in the external plexiform layer and mitral cell somas in the mitral cell layer. **B.** Averaged responses of tufted cells (upper) and mitral cells (bottom) to odor, ethyl butyrate (EB) at low (100x air dilution) and high (10x air dilution) concentrations without (black) and with (blue) an optogenetic pulse delivered at different latencies from the onset of inhalation (mean of 5 repetitions for 3 tufted cells and 4 mitral cells in a mouse). **C.** Average amplitude of D-MTCs (deep mitral/tufted cells) responses to a light pulse as a function of pulse timing in a sniff cycle without odor (black) and with the presentation of EB (mean of 5 repetitions for 7 D-MTCs, error bars are ± 1 SEM). . **D** Summary cartoon illustrating the expected. Transformation from olfactory sensory neurons (OSN) to different layers of the olfactory bulb (external tufted (eT), tufted (T), and mitral (M) cells. It illustrates that the eT cells follow the presynaptic signal, while tufted and mitral cells are subject to lateral inhibition. Additionally, it shows that in our observed data, there is less temporal filtering in tufted cells compared to mitral cells. (All presented data collected from mouse line: OMP-ChR2 x Thy1-GCaMP6f).

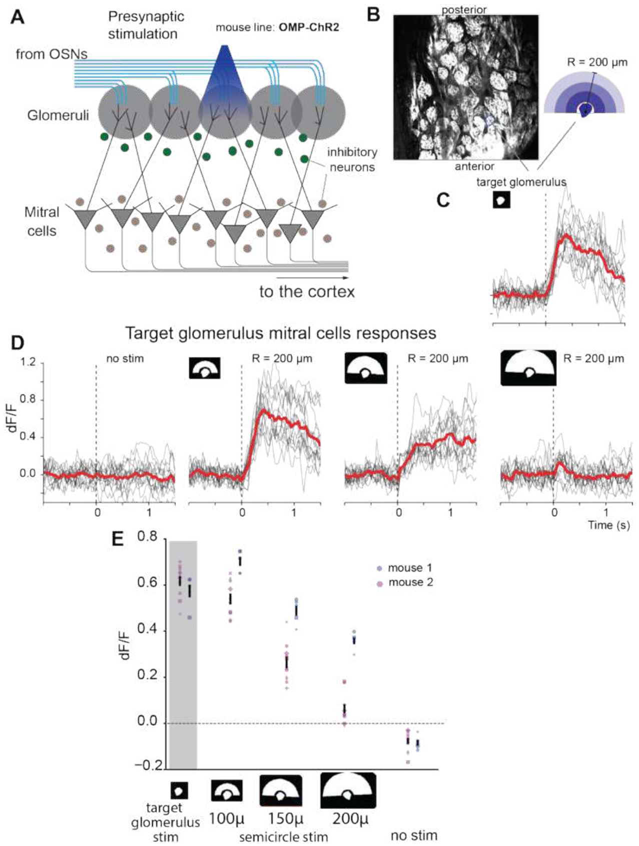

Figure S13: Lateral Inhibition in the Olfactory Bulb. A. Optogenetic stimulation of the OB in the OMP-ChR2 mice, where ChR2 is expressed in axons of OSNs. **B.** *Left:* 2-photon imaging of the glomerular layer. *Right:* Schematics of stimulation of a T-glomerulus and a semicircular area around this glomerulus with different radii. Semicircular stimulated area was positioned posterior to the T-glomerulus in order not to activate additional axons for of passage for T-glomerulus. **C.** Average (red) and individual (gray) activity traces of mitral cells connected to the T-glomerulus in response to optogenetic stimulation of the T-glomerulus (n= 10 cell, averaged across 8 trails, stimulation pulse: 20 mW/mm2, duration: 10 ms). **D.** Responses of the same cells to stimulation with the same parameters as in **C** for different size of the stimulated area. **E.** Amplitude of mitral cell responses for 2 mice (mouse 1: 5 cells, mouse 2:10 cells) for different stimulation regimes: only T-glomerulus stimulation, stimulation of T-glomerulus and semicircular area posterior to T-glomerulus with different radii, and no stimulation.

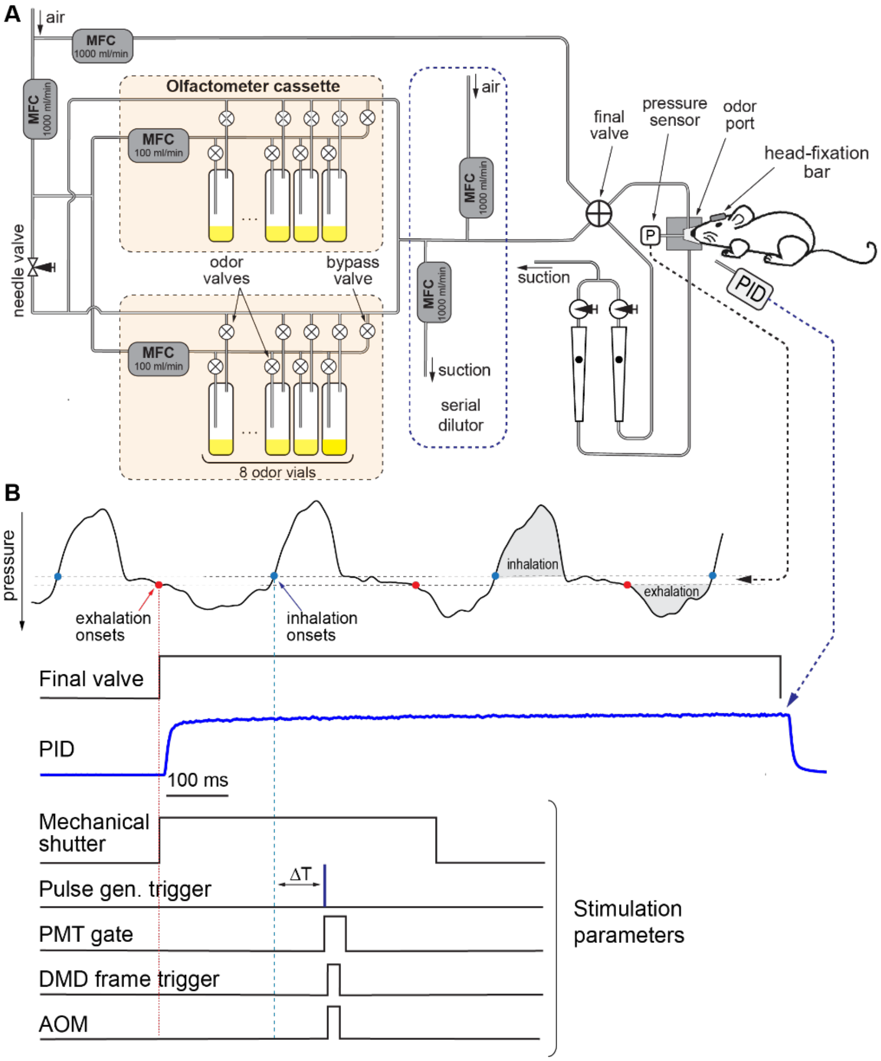

Figure S14. Odor delivery system and experimental timing A. Odors were delivered using a multi-cassette air dilution olfactometer. Each cassette had 8 odor vials, 16 odor valves, a mass flow controller (MFCs, 100 mL/min) and a normally open bypass valve. Total flow through both cassettes was controlled by a 1,000 mL/min MFC. To prepare an odor presentation, a pair of valves for a single odor vial was opened and the bypass valve was closed. The odorized air flow (1,000 ml/min) was first directed to the exhaust via the final valve (FV), while a clean air stream 1000 mL/min controlled by an additional MFC was delivered to the odor port. After approximately ~2 sec of flow stabilization, the FV redirected the odorized air to the odor port and the clean air to the exhaust. At the end of odor presentation, the final valve switched back, delivering clean air to the odor port. The concentration was controlled by the ratio of the MFC flow rates. To deliver a mixture, two vials from different cassettes were opened simultaneously. The olfactometer was equipped with a serial diluter, allowing further dilution of the odor concentration up to 20-fold. The airflow from the FV was directed to the odor port and then sucked away through the exhaust line, and both lines were balanced to minimize pressure buildup. The odor port was equipped with a pressure sensor to measure the mouse's sniff pattern and used to calibrate the airflows. **B.** The temporal profile of signals related to experimental control. The sniff pattern was continuously monitored by a pressure sensor. The FV was triggered by the onset of exhalation, so that the odor concentration was stabilized before the onset of the first inhalation. This was tested by measuring a temporal profile of odor concentration in the odor port using photo-ionization detector (PID). A TTL pulse was generated with a temporal offset, ΔT after the inhalation onset. The stimulation control consisted of PMT gating, DMD frame trigger, and AOM pulses. Photostimulation was delivered while gating the PMTs to prevent damage and saturation. The PMT gating signal began 2 ms earlier and ended 2 ms later to ensure gating.
